# Tracing 25 years of employee agility research: the missing links and future directions

**DOI:** 10.3389/fsoc.2026.1735781

**Published:** 2026-03-03

**Authors:** Genta Ramzuni, Rano Kartono Rahim, Mohammad Hamsal, Asnan Furinto, Willy Gunadi

**Affiliations:** Department of Management, BINUS Business School Doctor of Research in Management, Bina Nusantara University, Jakarta, Indonesia

**Keywords:** bibliometric analysis, digital competence, employee agility, employee resilience, innovation, psychological empowerment

## Abstract

Over the past two decades, the growing turbulence of the VUCA era, accelerated by the COVID-19 pandemic and digital transformation, has compelled organizations to develop agility as a vital capability for survival and adaptation. However, while organizational agility has received considerable scholarly attention, research on employee agility as its micro-foundation remains limited and fragmented, leaving substantial gaps in understanding the individual-level factors that shape organizational adaptability. This study presents a comprehensive bibliometric review of research on employee agility over the past 25 years, aiming to map its intellectual, conceptual, and social structures, identify gaps in the existing literature, and outline future research directions. The analysis encompasses 319 publications indexed in Scopus and Web of Science, spanning the period from 2000 to 2025. Using Biblioshiny and VOSviewer, the study examines publication trends, citation networks, thematic structures, and emerging research frontiers. The results reveal a sharp increase in publications since 2020, driven by the COVID-19 pandemic and digital transformation pressures. Three main clusters dominate the field: (1) organisational responses to change and disruption, (2) HRM practices and individual-level mechanisms, and (3) collaboration, knowledge sharing, and digital platforms. Despite these developments, significant gaps remain, including the limited integration of leadership, weak linkage between employee agility and innovation, the divide between HR-driven and digital-driven agility, and the underrepresentation of studies in the public sector and Southeast Asia. By synthesising fragmented insights across two major databases, this study highlights employee agility as a micro-foundation of organisational agility and proposes future research directions focusing on leadership, digital competence, innovation, psychological factors, and public sector contexts. These contributions position employee agility as not only a short-term adaptive capability but also a strategic enabler of innovation and sustainability in dynamic environments.

## 1 Introduction

Over the past two decades, the world has entered an era of volatility, uncertainty, complexity, and ambiguity (VUCA), leading to widespread changes and disruptions across all aspects of life. The COVID-19 pandemic in 2020–2021, which led to a slowdown in the global economy, ushered in a “new normal” way of life (Ajgaonkar et al., 2022; Rauf et al., 2023; Yesudhas et al., 2021). At the same time, the digital revolution has continued unabated, marked by the growing use of artificial intelligence (AI), the Internet of Things, and automated work systems that have transformed how people work and interact (Jindal et al., 2021). These conditions have led organizations—as economic entities, social structures, and dynamic systems (Selznick, 1948) to be required not only to respond and adapt to change, but also to collaborate and innovate in a dynamic environment proactively. In other words, organizations are expected to possess agility as a vital capability to survive in rapidly changing environments.

Given that organizations are essentially run by individuals who execute the strategies and policies formulated by the organization, an agile organization is ideally operated by agile human resources. Therefore, along with the growing need to cultivate agility at the organizational level, academic and practitioner attention has also begun to shift toward agility at the individual level as a micro-foundation of organizational agility in both private and public sectors. Individual-level agility—commonly defined as proactive, adaptive, and resilient behavior (Alavi et al., 2014; Chong and Zainal, 2024; Lin et al., 2025; Loghmani et al., 2023; Pitafi et al., 2025d; Zhang et al., 2022), is seen as a determinant of how organizations respond to social and environmental dynamics. In the public sector context, employee agility has been defined as the collaborative behaviors of public employees in responding to crises and environmental changes (Albrecht, 2024). Team-level agility (workforce agility) has likewise been shown to mediate the relationship between organizational digitalization and performance (Sameer, 2024), as well as the relationship between leadership and performance (Varshney and Varshney, 2024). However, despite the burgeoning attention to the urgency of employee agility as a micro-foundation of organizational agility, employee agility itself has yet to become a major focus of research among academics (Nguyen et al., 2024).

Although research on employee agility has grown rapidly and significantly in recent years, there is an increasing risk of conceptual overlap and fragmented inquiry. The existing literature is structurally dispersed across journals, themes, and disciplinary perspectives, limiting scholars’ ability to discern dominant research trajectories and cumulative patterns. Moreover, employee agility and workforce agility are frequently discussed alongside similar constructs such as leanness, flexibility, and adaptability, resulting in blurred conceptual boundaries (Christofi et al., 2021; Nguyen et al., 2024). As a result, many studies focus on specific antecedents, outcomes, or contextual applications of agility, making it difficult to obtain a comprehensive understanding of how the field has evolved as a whole (Nguyen et al., 2024). Therefore, a bibliometric analysis is necessary not only to map the development of employee agility research over more than two decades but also to systematically synthesize an increasingly fragmented body of literature, clarify overlapping conceptualizations, and identify comparatively underrepresented dimensions and contexts that can inform future research agendas.

Amid the growing urgency to cultivate employee agility as a microfoundation of an organization’s dynamic capabilities, research on this topic remains limited and fragmented, and thus has yet to provide a comprehensive view for scholars or practitioners. Correspondingly, Nguyen et al. (2024), through a systematic review of 249 studies showed that agility contributes significantly to organizational performance, but most research remains more focused on the organizational level than on the individual level. Several previous studies have used a bibliometric approach to map the development of agility research by classifying the dominant antecedents, mechanisms, and outcomes in the literature. Among them, Reyes et al. (2024) conducted a comprehensive bibliometric and content analysis of learning agility research. While this makes an important contribution to understanding agility as an individual learning capability, it represents only one dimension of employee agility. Therefore, learning agility does not fully capture employee agility as a broader capability encompassing adaptive, proactive, and resilient behaviors in responding to changes in the work environment. Meanwhile, research specifically mapping research on workforce agility was conducted by Alviani et al. (2024) using a systematic literature review method. This study focused on an inventory and thematic classification of research on workforce agility through 2024, thereby providing a comprehensive overview of the positioning of employee agility variables and the research methodologies used in previous studies. However, this study does not fully understand the intellectual structure, conceptual evolution, and cross-level interrelationships that shape employee agility as an adaptive, proactive, and resilient capability.

Based on the above background, this bibliometric study aims to systematically map the development of employee agility research over the period 2000–2025; identify the most influential sources, authors, and documents; and trace the intellectual, social, and conceptual structures on the field through citation and co-word analysis. Accordingly, this study addresses the following research questions: (RQ1) How has employee agility research evolved over time in terms of publication trends, influential sources, and geographic distribution? (RQ2) What are the dominant themes and intellectual structures characterizing employee agility research, as revealed through science mapping? (RQ3) How is employee agility conceptualized across the main thematic clusters? And (RQ4) Which conceptual dimensions, contextual settings, and research perspectives remain comparatively underrepresented within an increasingly mature body of employee agility research?

By explicitly focusing on employee agility as a micro-foundation of organizational agility, this study contributes to the literature by providing a comprehensive, longitudinal mapping of the field over 25 years. Using Biblioshiny and VOSviewer, the analysis integrates performance indicators with science mapping techniques to support systematic synthesis and theory development. In doing so, this study provides a structured basis for identifying underexplored areas and formulating a future research agenda with both theoretical and practical relevance.

The structure of this paper consists of an Introduction that presents the background and justification for a bibliometric analysis on the topic of employee agility, followed by sections detailing the conceptual framework, methodology of the bibliometric analysis, the results and findings of the analysis, a discussion of the results, and finally the conclusion along with research limitations and future research directions.

## 2 Conceptual framework

Most research on employee agility has focused on agility at the organizational and team (workforce) levels. There have been few studies centered on agility at the individual level, even though this individual dimension began to appear in the literature around 2020. This gap is unfortunate, considering that agility at the organizational and team levels is fundamentally built upon the agility of the personnel or individual members within those entities. Employee agility at the individual level refers to an individual’s capacity to respond proactively to dynamics in the work environment, the ability to adapt quickly, flexibility, and initiative in facing the complexity of organizational tasks (Raut et al., 2022). Team-level agility (workforce agility) has also been shown to mediate the relationship between an organization’s digitalization and its performance (Sameer, 2024), as well as to mediate the relationship between leadership and performance (Varshney and Varshney, 2024).

A notable contribution in this area is the work by Petermann and Zacher (2021) who developed a behavioral taxonomy of employee agility using qualitative methods, thereby depicting employee agility as concrete behaviors rather than merely an abstract psychological concept. Another recent study by Pitafi et al. (2025a, 2025c) defines workforce agility as an individual’s ability to respond to unexpected environmental changes quickly and effectively. Employee agility was measured with three main dimensions, namely the ability to actively anticipate and handle change (proactivity), the ability to adjust one’s behavior and work approach to new conditions (adaptability), and the capacity to withstand pressure or stress when facing change (resilience) (Pitafi et al., 2025a, 2025c). Other studies have applied similar dimensions to define and measure employee agility while examining various antecedents influencing it, for example, Chong and Zainal (2024) and Varshney and Varshney (2024). Collectively, these studies underscore the importance of agility at the individual level as a building block or predictor of organizational agility, which in turn influences an organization’s strategies to survive and perform optimally during periods of change, as agility is an enabler of innovation.

Early research on employee and workforce agility has mainly relied, directly or indirectly, on the Resource-Based View (RBV), which considers human skills, flexibility, and expertise as valuable organizational resources that enhance performance and competitive advantage. From this perspective, agility-related traits are often viewed as relatively fixed assets embedded in employees or the workforce. However, several scholars have pointed out that RBV has limited explanatory power in dynamic and turbulent environments because it downplays innovation, managerial orchestration, and the ongoing reconfiguration of human resources. Ajgaonkar et al. (2022) explicitly note that early studies on workforce agility were largely grounded in the RBV and only loosely connected to dynamic capability theory, calling for a shift in theory that better captures the changing nature of work and organizational transformation. From a dynamic capability perspective, workforce and employee agility are individual-level dynamic capabilities that are prerequisites for organizational-level agility (Flavio et al., 2024). The framework of Dynamic Capabilities Theory explains how an agile workforce can become one of the organizational capabilities used to integrate, build, and reconfigure competencies in response to change, so that agile employees are viewed as organizational capabilities that will support strategic agility (Sharma et al., 2024).

Within DCT, agility is a capability that enables organizations to sense environmental changes, seize new opportunities, and continuously reconfigure resources and routines. Ajgaonkar et al. (2022) and Flavio et al. (2024) characterize workforce agility as a form of dynamic capabilities, grounded in micro-foundations such as sensing, seizing, and ongoing renewal, and supported by leadership decisions and HRM practices. Similarly, Jooss et al. (2024) contend that strategic and workforce agility cannot be achieved through static talent pools alone but require dynamic processes such as skills matching, workforce reconfiguration, and resource fluidity, emphasizing that agility results from coordinated human and managerial efforts. In line with this theoretical development, modern studies increasingly view employee and workforce agility as part of a dynamic capabilities portfolio rather than an outcome, which includes overlapping aspects such as adaptability, flexibility, resilience, and proactivity, which collectively support organizational change and innovation (Atanassova et al., 2025; Dubey et al., 2023; Flavio et al., 2024; Jafari-sadeghi et al., 2022). Human resource management, leadership, and learning practices are also seen as mechanisms to build agility as micro-foundation dynamic capabilities, since without agile employees, the organizational capability to sense, seize, and transform is difficult to implement (Ajgaonkar et al., 2022; Flavio et al., 2024; Sharma et al., 2024).

Other contemporary employee agility research increasingly acknowledges the value of integrative perspectives that allow multiple explanatory mechanisms to coexist, capturing how agility is enacted at the individual level and how it scales across teams and organizations. Those studies proposed several individual-related antecedents of organizational agility, such as individuals’ perceptions (Ludviga and Kalvina, 2024), trust toward organization (Jager et al., 2022), talent management (Aldabbagh, 2023), and dynamic capabilities of the organization (Zhang et al., 2025). It reflects a broader evolution in how agility is understood, as a multifaceted phenomenon shaped by individual cognition, behavioral responses, interpersonal interactions, and the surrounding context. From this perspective, employee agility is not tied to a single theoretical tradition, but can be examined through a range of complementary viewpoints that shed light on leadership influences, organizational settings, and the psychological dynamics underlying individual adaptability.

## 3 Methodology

**Figure 1:**
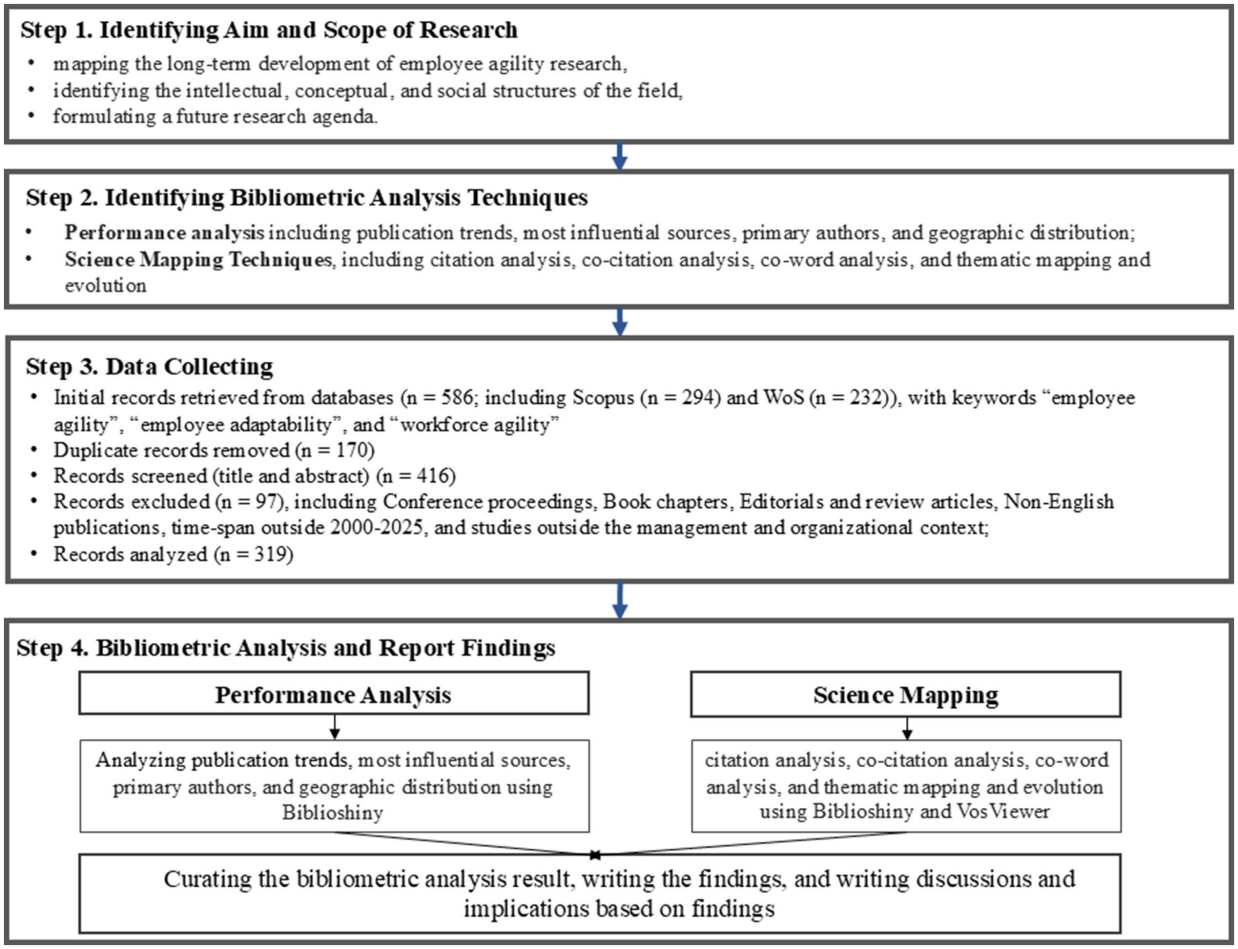
The steps of bibliometric analysis. This figure shows how we conducted the bibliometric analysis, including the aim, techniques, data collecting and numbers of articles involved, and bibliometric report and findigs.

This study employed a bibliometric analysis method to systematically examine research on employee agility and its development over the last 25 years (2000–2025). This research approach was chosen because it can provide a quantitative overview of publication trends, knowledge networks (both conceptual and intellectual), and thematic evolution of a research topic or field—especially one that remains fragmented. The bibliometric analysis in this article was conducted in two stages, namely performance analysis and science mapping. The performance analysis phase aims to identify trends in employee agility publications based on annual publication counts, authors, institutions, countries, and the most productive and influential publication sources. This analysis was based on results generated by Biblioshiny. Meanwhile, the science mapping focuses on uncovering the intellectual, social, and conceptual relationships among publications. This stage was carried out through co-citation analysis to identify the most influential works (Chen et al., 2016), co-word analysis to map core research themes (Kocaman and Kanbach, 2024), and thematic mapping and trending topics analysis to trace thematic evolution over the 25 years. Together, these two analysis stages underscore that bibliometric analysis serves not only to describe the productivity and impact of publications, but also to reveal the knowledge structure and emerging research directions in a field (Donthu et al., 2021). In line with Passas (2024) and Donthu et al. (2021), the bibliometric analysis of employee agility in this study was carried out in several key stages: defining the research objectives, extracting bibliographic data, cleansing and standardizing the data, analyzing the data, and visualizing and interpreting the results. The details of the analysis stages are depicted in the Donthu Diagram presented in Figure 1.

To analyze the collected dataset, this study utilized the Biblioshiny application (from the R-package Bibliometrix) and VOSViewer. Biblioshiny was used to support descriptive analysis of the dataset, such as publication trends, influential sources and authors, as well as thematic mapping and thematic evolution of employee agility research. Meanwhile, VOSViewer was used to construct co-citation and co-word network maps, allowing exploration of relationships or networks among research themes. VOSViewer was chosen because it is one of the most widely used tools in bibliometric analysis for illustrating interconnections among research concepts in the form of intuitive and interactive network maps (Bukar et al., 2023).

The data sources for this study were obtained from the Scopus and Web of Science databases, which are widely recognized as trusted sources of indexed, reputable international scholarly publications and which offer broad coverage. The search was conducted using the main keyword “employee agility” and related terms such as “workforce agility” and “employee adaptability.” This initial search identified 294 documents in the Scopus database and 232 documents in the Web of Science database with titles, keywords, or abstracts containing these terms. The retrieved data were then cleansed using parameters for publication timeframe, document type, field of study, and language. The publication timeframe was set from 2000 to 2025 to ensure the bibliometric analysis captured the research’s long-term trends and development. To maintain methodological consistency and analytical precision, this study limits the dataset to peer-reviewed journal articles published in English. Journal articles offer standardized, reliable bibliographic metadata, which is crucial for citation-based and co-word analyses. English was selected as the language of analysis because it is the primary language of international scholarly communication and facilitates meaningful comparisons across countries and disciplines. This approach adheres to established bibliometric review guidelines and helps minimize linguistic and structural differences that could otherwise distort science mapping results. While this approach enhances analytical consistency, it may underrepresent relevant studies published in other languages or dissemination formats. The subject areas of the publications were also restricted to business and management, psychology, arts and humanities, social science, computer science, and decision science. Other fields were excluded from the dataset because, aside from being small in number, they were not closely related to the field under review or mapping. For example, the search query used in Scopus was:

TITLE-ABS-KEY (“employee agility” OR “workforce agility” OR “employee adaptability”) AND PUBYEAR > 1999 AND PUBYEAR < 2026 AND (LIMIT-TO (SUBJAREA, “BUSI”) OR LIMIT-TO (SUBJAREA, “PSYC”) OR LIMIT-TO (SUBJAREA, “ARTS”) OR LIMIT-TO (SUBJAREA, “SOCI”) OR LIMIT-TO (SUBJAREA, “COMP”) OR LIMIT-TO (SUBJAREA, “DECI”)) AND (LIMIT-TO (DOCTYPE, “ar”)) AND (LIMIT-TO (LANGUAGE, “English”)).

Of the 294 records found in Scopus, 197 met the criteria and were eligible for inclusion in the analysis. These 197 records were then combined with the 232 documents from Web of Science and cleansed by removing duplicate entries, yielding a final dataset of 319 documents to be analyzed.

## 4 Results

The analysis of the collected dataset revealed findings that support research on employee agility. In this section, these findings are organized and presented as Performance Analysis and Science Mapping, providing comprehensive overview of employee agility research over the past 25 years.

### 4.1 Performance analysis

#### 4.1.1 Trend of publications per year

**Figure 2:**
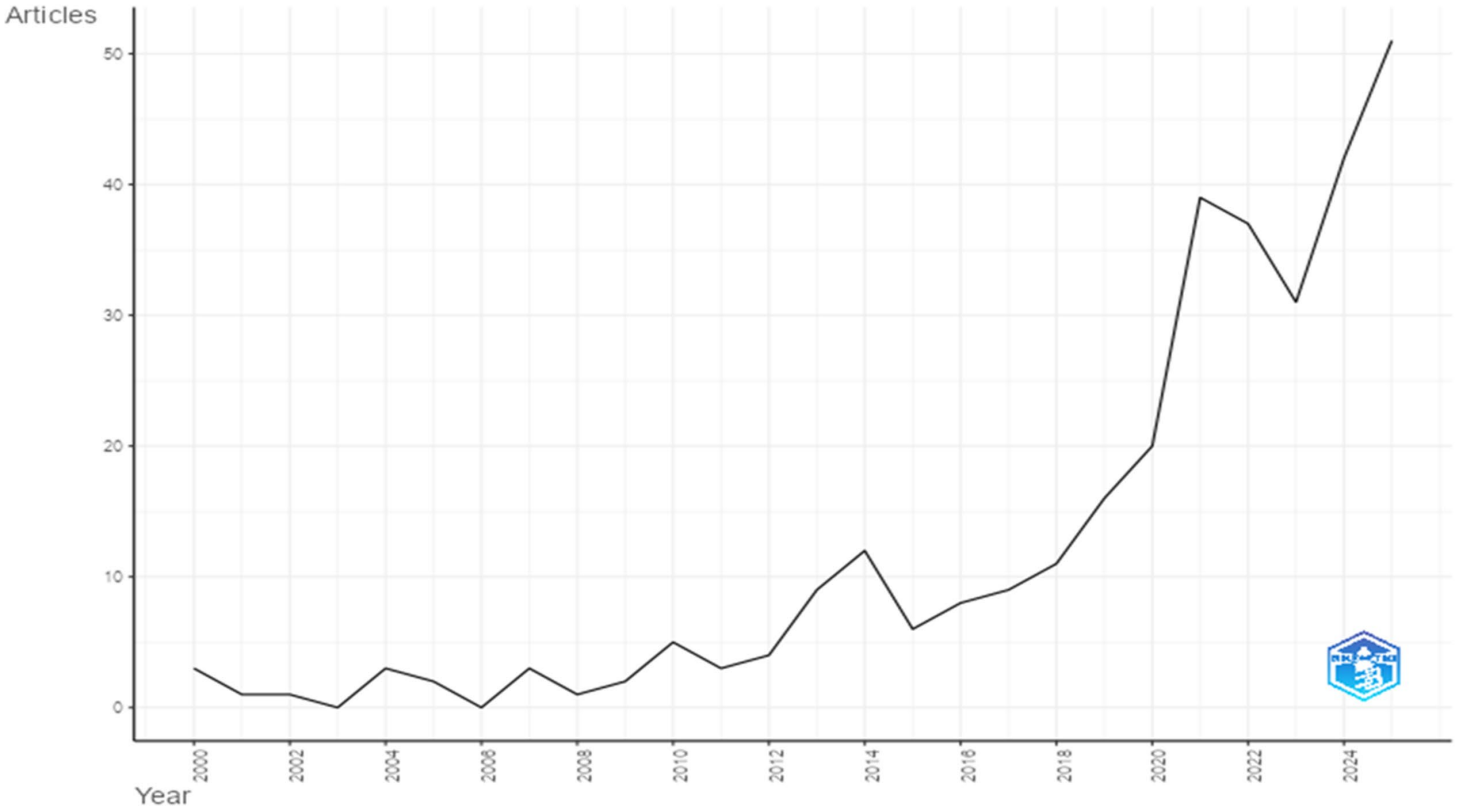
Annual scientific production. This figure shows the trend in the number of articles on employee agility over the past 25 years (Year 2000 – 2025).

Bibliometric analysis of the development of employee agility research over the past 25 years was conducted by mapping the annual output of articles on employee agility, to examine how academic interest in this topic has evolved. The trend in the number of articles on employee agility over the past 25 years is shown in Figure 2. The processed data on the annual trend of employee agility publications from 2000 to 2005 indicate an average growth of about 12% per year. The graph in Figure 1 also shows that publications on employee agility from 2000 to 2018 remained below 10 articles per year, except 2014, which saw a slight uptick to 12–13 articles. A surge in the number of employee agility studies occurred in 2019 and continued through 2024, during which the output of articles on this topic rose to more than 40 per year. This trend was likely driven by the COVID-19 pandemic in 2019, which caused disruptions in nearly every aspect of life and forced the world to live in a new normal era. As a result, agility—closely tied to the ability to respond and adapt to change—became a topic of heightened interest in academia.

The increase in studies on employee agility since 2020 reveals a growing academic focus on employees’ ability to adapt to increasingly dynamic and uncertain work environments. This rise in publications during the post-COVID-19 period suggests that employee and workforce agility are being recognized more as strategic capabilities. They are viewed not only as essential for supporting operational and technical work aspects but also for addressing employees’ psychological wellbeing. In line with previous empirical findings, agility is understood as a mechanism that helps organizations maintain performance while better preparing for future crises (Raut et al., 2022; Srivastava and Gupta, 2022). Furthermore, the growing attention to employee adaptive capabilities is also related to the acceleration of digital transformation and the adoption of work technologies, which demand flexibility and continuous learning at the individual level (Maran et al., 2022; Sony and Mekoth, 2022b). In the digital era, agility at the individual level, encompassing adaptability, proactivity, and resilience, is known to be a capability that can influence performance, innovation, and an organization’s ability to adopt new management models (Alviani et al., 2024). Therefore, the increase in employee agility research since 2020 reflects a shift in conceptual attention to agility as a dynamic capability. This increase was followed by conceptual differentiation, which will be further discussed in the next section through a science mapping analysis.

#### 4.1.2 Most relevant sources and cited documents

**Figure 3:**
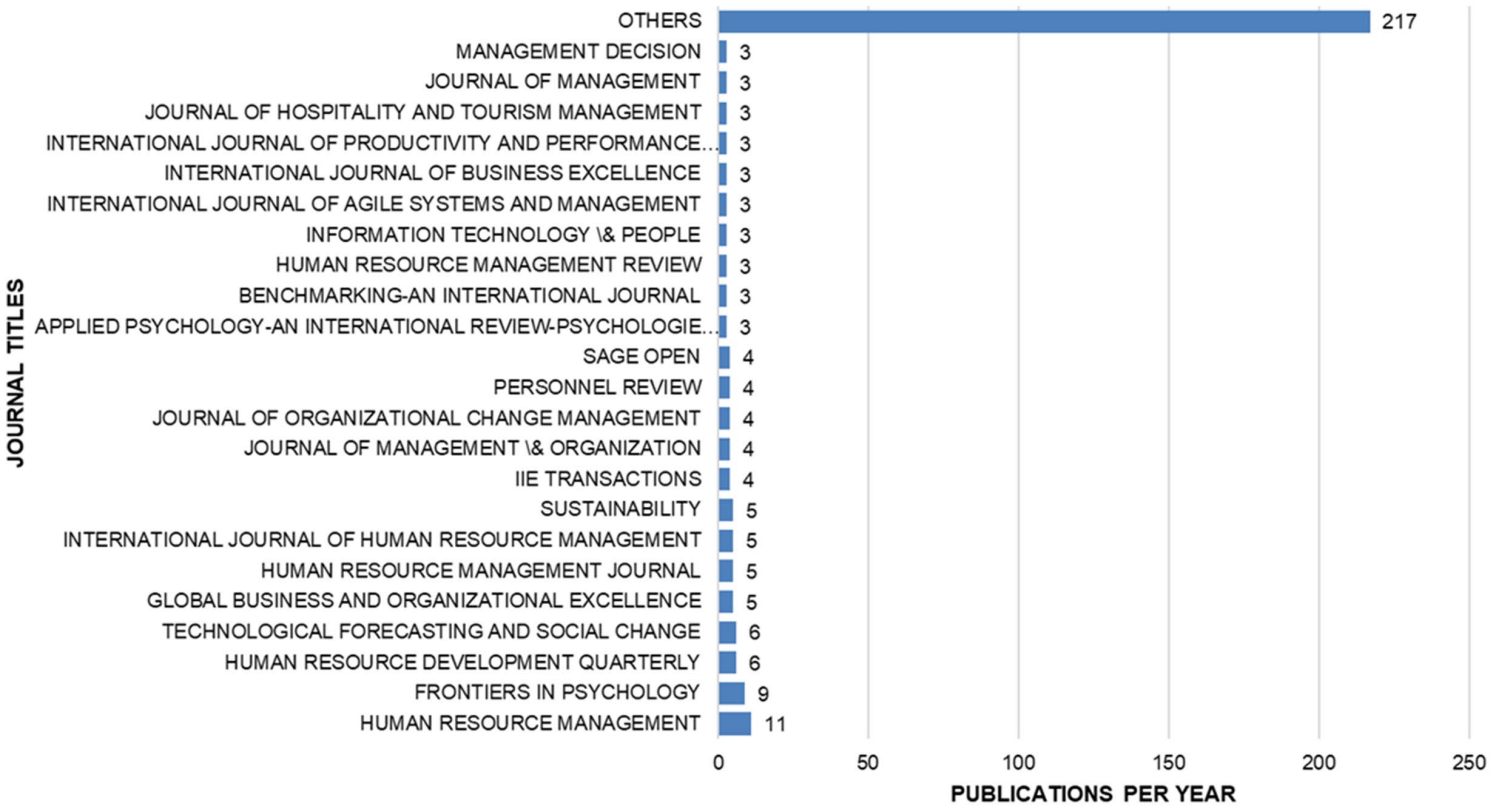
Most relevant sources in employee agility research. This figure shows the publication sources and articles that have published the most content on the theme of employee agility from the years 2000 – 2025.

Bibliometric analysis was also conducted on the publication sources and articles that have published the most content on the theme of employee agility. Analyzing these data is important to provide an overview of the distribution of employee agility research across disciplines. The data on these sources are presented in Figure 3. The results shown in Figure 3 indicate that the majority of publications related to employee agility have been published in the journal Human Resource Management (11 articles), followed by Frontiers in Psychology (9 articles), Human Resource Development Quarterly (6 articles), and other journals focused on social science and psychology. Notably, journals outside the social sciences, such as Information Technology and People, have also published work on employee agility. Other articles are scattered across various journals that focus on general management and decision-making. This diversity of journal outlets suggests a cross-disciplinary trend in research on employee agility, spanning work psychology, management, and even technology-oriented disciplines.

**Figure 4:**
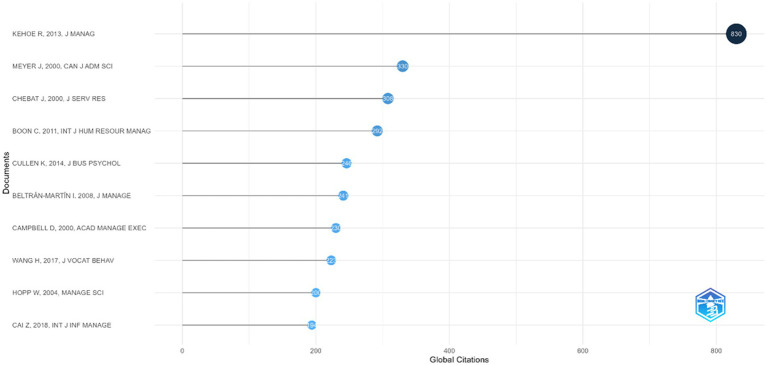
Most global cited documents regarding employee agility (2000-2025). This figure shows the most highly cited articles globally.

Meanwhile, an analysis of the most highly cited articles was also performed, since a high number of citations provides insight into which research works serve as key intellectual foundations and main references in employee agility research. The documents that are most cited in employee agility studies from 2000 to 2025 are shown by Figure 4. These data showed that the three most-cited documents on employee agility are an article by Rebecca R. Kehoe and Patrick M. Wright titled “The Impact of High-Performance Human Resource Practices on Employees’ Attitudes and Behaviors,” with 830 citations. This article examines the relationship between employees’ organizational citizenship behavior and their perceptions of human resource management practices, as well as the factors that influence that relationship (Kehoe and Wright, 2013). Another highly cited article is by Jean-Charles Chebat and Paul Kollias titled “The Impact of Empowerment on Customer Contact Employees’ Roles in Service Organizations,” with 330 citations. This article focuses on how employees in the financial services sector cope with role conflict and role ambiguity, involving factors such as adaptability, self-efficacy, and job satisfaction (Chebat and Kollias, 2000). The next most-cited article is by Wang et al. (2017) with 280 citations; this work focuses on employee adaptability in the form of job crafting, which is influenced by transformational leadership. The subsequent highly cited publication is by Cullen et al. (2014) with 246 citations; this article primarily discusses how employees’ perceptions of change and organizational support influence job satisfaction and performance. Another frequently cited study is by Beltrán-Martín et al. (2008) with 241 citations, which was one of the early articles explicitly linking human resource management practices with workforce agility. Additionally, an article by Hopp et al. (2004) with 200 citations, connects agility with flexibility in management.

An analysis of the most relevant sources and most-cited documents shows that research on employee agility remains strongly rooted in human resource management, where employee agility is viewed as a behavior arising from employee talent development, leadership, and job design. Employee agility, as a product of human resource management, then becomes a flexible behavioral mechanism that allows employees to respond effectively to organizational demands, especially those related to performance improvement. Therefore, within this framework, employee agility links managerial systems and organizational performance to the organization’s adaptive capacity, of which employee agility is the micro-foundation. The dominance of this approach indicates that HRM provides a key intellectual foundation for early research on employee agility, supporting further conceptual expansion in later phases.

#### 4.1.3 Most relevant author and corresponding authors’ countries

**Figure 5:**
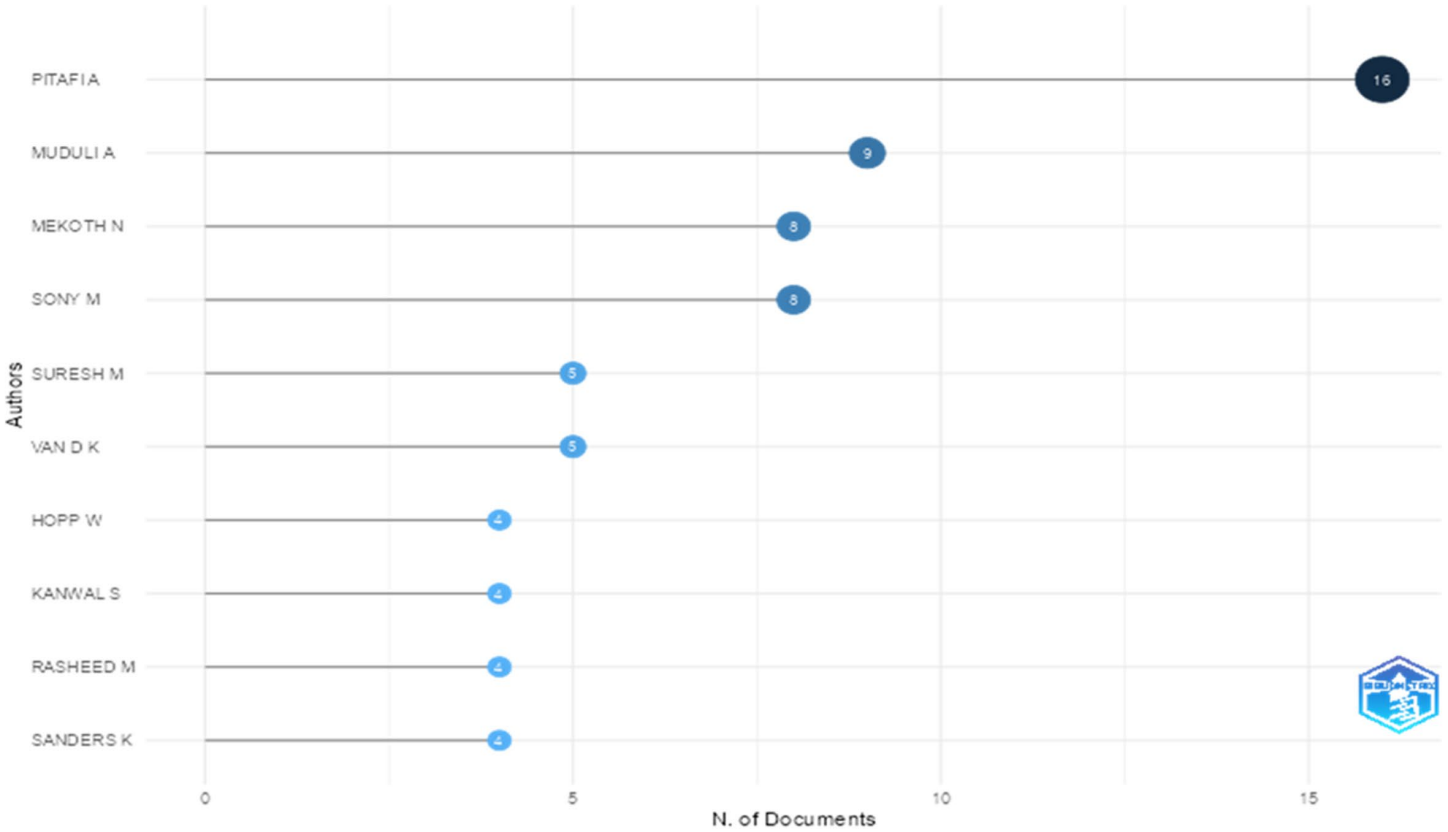
Most relevant author(s) of article about employee agility (Years 2000 – 2025). This figure illustrates who the key figures are that have consistently contributed to building and expanding the literature, and the disciplinary loci that are driving growth in this research topic from 2000 – 2025.

In addition to publication sources and influential articles, the bibliometric analysis also identified the most prolific authors in the field of employee agility. This helps illustrate who the key figures are that have consistently contributed to building and expanding the literature, and it also reflects the disciplinary loci that are driving growth in this research topic. The most relevant authors in the field of employee agility research are shown in Figure 5. This figure shows that Abdul Hameed Pitafi is the author with the highest number of articles on employee agility (16 articles). This finding underscores that Pitafi is a key figure in employee agility research, particularly on issues related to digitalization and digital innovation. Other authors such as Nandakumar Mekoth, Ashutosh Muduli, and Michael Sony, each with seven articles, also play important roles in extending the agility literature in the context of human resources and organizational behavior.

Articles published by Pitafi are known for their focus on research linking the role of enterprise social media with employee agility behavior, where enterprise social media can influence employee agility through higher quality communication, the formation of psychological safety, knowledge transfer, knowledge sharing, and more visible communication (Liu and Soomro, 2025; Pitafi et al., 2019, 2020; Pitafi, 2025; Pitafi et al., 2025c; Pitafi and Ren, 2021; Wei et al., 2020). In addition, in several of his studies, Pitafi also highlighted problems in the context of work implementation, such as conflict, stress, and work structure that can be related to employee agility (Pitafi, 2025; Pitafi et al., 2018, 2020), as well as the relationship between employee agility and digital competence and employee creativity (Lai et al., 2021; Pitafi et al., 2020; Rasheed et al., 2023; Wei et al., 2020). Meanwhile, the articles written by Nandakumar Mekoth together with Michael Sony mostly have a main focus on frontline employee adaptability in the service sector, both in its measurement, its relationship with the psychological side and organizational support, as well as the relationship between employee agility and technological skills possessed by employees (Sony and Mekoth, 2012, 2014, 2015, 2016a, 2017a, 2017b, 2019, 2022). Meanwhile, Ashutosh Muduli mostly writes articles about employee agility in the scope of learning agility and knowledge transfer, and the relationship between employee agility and employee psychological conditions or psychological empowerment, as well as their digital capabilities or abilities (Ghosh and Muduli, 2021; Muduli, 2016, 2017; Muduli and Choudhury, 2025; Muduli and Pandya, 2018; Srigouri and Muduli, 2024a).

An analysis of the most cited authors shows that the main intellectual contributions to employee agility research have developed through several distinct yet complementary thematic areas. Pitafi’s work mainly links employee agility to the digital environment and social interactions, especially through the role of enterprise social media in shaping communication quality, psychological safety, and knowledge sharing and transfer processes. Meanwhile, research by Sony and Mekoth focuses primarily on the adaptability of frontline employees in the service industry, emphasizing the importance of psychological wellbeing, organizational support, and technological skills in promoting agility. Muduli’s contribution, on the other hand, places employee agility within a framework of learning agility and psychological empowerment, highlighting the significance of individual learning, empowerment, and digital skill development. Overall, this pattern indicates that the most influential literature on employee agility has moved beyond traditional HRM methods toward a more multidimensional understanding that includes digital, psychological, learning, and workplace contexts.

**Figure 6:**
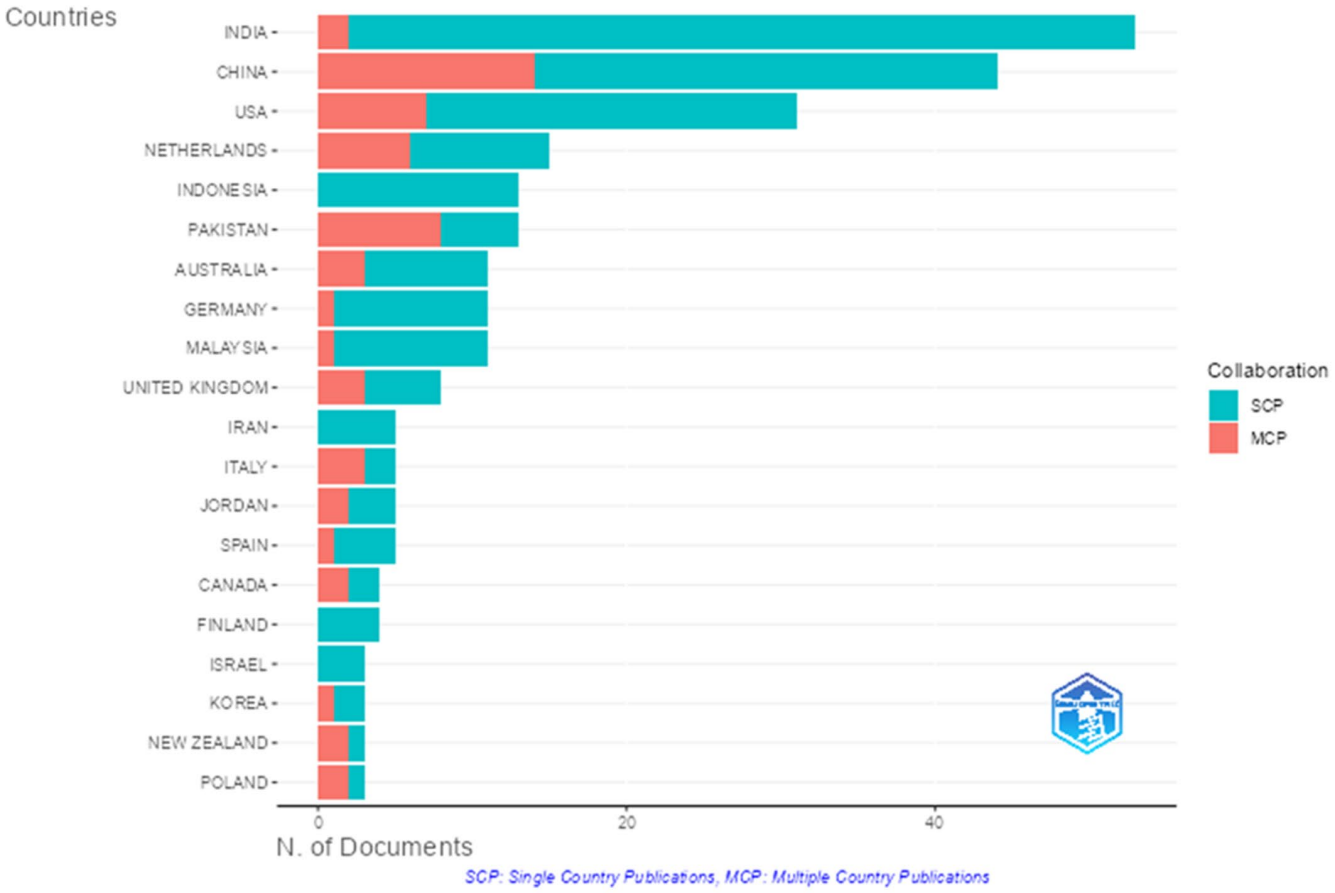
Employee agility article’s corresponding authors’ countries (Years 2000-2025). This figure shows the distribution of the countries of origin of corresponding authors in employee agility research from the years 2000 - 2025.

An interesting finding from this analysis is that most productive authors come from an Asian background, especially India and Pakistan, which may influence the research environment. The distribution of countries of origin for corresponding authors in employee agility research is shown in Figure 6. The figure reveals that the largest number of employee agility articles was written by authors from India, with over 40 publications. Most of the publications by India-based authors were single-country studies (domestic research), rather than international collaborations involving multiple countries. Corresponding authors from China and the USA also contributed a significant number of articles, with each country producing over 20 and over 10 publications, respectively. Similar to India, research by authors in China and the USA more often took the form of domestic studies rather than cross-country collaborations. A similar trend is seen among corresponding authors from Malaysia, Australia, the Netherlands, Germany, and several other nations. In Indonesia, nearly all publications in this field have been conducted domestically. However, a different pattern appears in countries like Pakistan, Italy, Canada, New Zealand, and Poland, where corresponding authors more frequently engaged in cross-country research collaborations than purely domestic studies (even though the total number of publications from each of these countries is relatively small, fewer than 10 documents). This is notable because extensive domestic and international researcher networks can expand the impact and discussion on employee agility within the academic community.

The distribution of the most prolific and corresponding authors by country indicates that research on employee agility has primarily developed in an Asian context, especially in India and Pakistan. This pattern suggests that most empirical work on employee agility originates from organizational settings in developing countries and economies experiencing rapid change, where issues like workforce flexibility, digitalization, and human resource management are particularly relevant. Additionally, the dominance of domestic publications (single-country studies) in nearly all contributing countries implies that employee agility is mainly studied within specific national and institutional contexts. While some countries are more active in cross-border collaboration, international networks in employee agility research are generally limited. This highlights the contextual nature of the development of the employee agility literature and offers insights into the geographic and institutional factors shaping knowledge in this field.

#### 4.1.4 Most cited country

**Figure 7:**
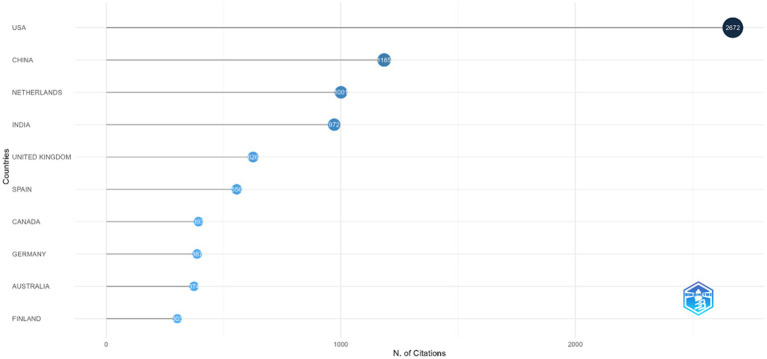
Most cited country in articles regarding employee agility (Years 2000 – 2025). This figure shows the countries that are cited the most in studies on employee agility.

To examine the centers of intellectual influence in employee agility research globally, a bibliometric analysis was conducted of the countries most frequently cited in studies on employee agility. Over the last 25 years, the status of the most cited countries as shown by Figure 7. The analysis identified that the United States (USA) has been cited 2,672 times in articles on employee agility, making it the most frequently cited country in the global research landscape. Meanwhile, China and the Netherlands are the second- and third-most-cited countries in employee agility research, with 1,185 and 1,001 citations, respectively. Notably, the data do not yet show any Southeast Asian countries with substantial citation counts on this topic, reflecting that research originating from those countries has not (yet) been prominent in the literature.

Identifying the countries most frequently cited in employee agility research is important since it indicates the research contexts considered representative and relevant to understanding employee agility globally. Thus, a high number of citations from a given country not only reflects academic influence, but also suggests that the social, economic, and organizational contexts of that country are deemed pertinent and illustrative for the global understanding of employee agility. The dominance of the United States, China, and Western European countries as the most-cited nations indicates that the development of employee agility has been heavily influenced by established organizational and managerial settings in the global literature. The high citation rates from these countries suggest that empirical findings and conceptual frameworks from these contexts are the main reference points for understanding and explaining employee agility. On the other hand, the limited number of citations to research from Southeast Asia indicates that contributions from these regions have not yet been widely integrated into mainstream discussions of employee agility. This pattern suggests that although research on employee agility is growing geographically, its intellectual structure remains relatively concentrated, with dominant ideas about employee agility mainly shaped by the organizational and institutional experiences of a few key countries.

#### 4.1.5 Most frequent words

**Figure 8:**
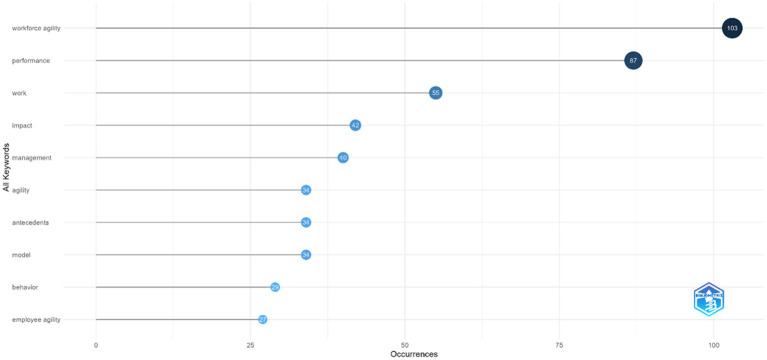
Most frequent word used in articles regarding employee agility (Years 2000 – 2025). This figure shows the most dominant keywords used by researchers in articles on employee agility from the years 2000 – 2025.

This study also explored the most frequently used keywords by researchers in articles on employee agility. By analyzing patterns of commonly used terms, we can identify the main focus areas within employee agility over the past 25 years. The results, showing the most common words in employee agility research from 2000 to 2025, are displayed in Figure 8. The analysis revealed that the keywords “performance” (27 times) and “work” (55 times) are frequently used in this field. Other common terms include impact, management, and behavior, indicating a shift toward viewing agility as proactive capabilities that enable individuals to actively adjust and shape their work. The distribution of frequently used keywords highlights a strong emphasis on performance-related outcomes and work contexts, suggesting that the literature predominantly frames the phenomenon in terms of its practical implications rather than as an isolated concept. The prominence of terms such as performance, work, and impact indicates that earlier studies have mainly focused on how adaptive and responsive capabilities lead to tangible organizational results. Meanwhile, the presence of keywords related to management, antecedents, models, and behavior reflects a growing scholarly interest in understanding the underlying mechanisms, behavioral processes, and organizational conditions through which these capabilities are developed and enacted. Overall, this pattern suggests an evolution of the research toward more integrated explanations that connect individual behavior, management practices, and organizational performance.

### 4.2 Science mapping

After the descriptive analysis that highlighted publication trends, sources, authors, and keywords, this section discusses the next phase: the science mapping analysis. Science mapping shows how the literature on employee agility is interrelated and has developed over time. Using co-citation and co-word analysis techniques, this mapping helps to uncover knowledge networks, the main thematic clusters, and the evolution of research directions that form the conceptual foundation of this field.

#### 4.2.1 Co-citation network

**Figure 9:**
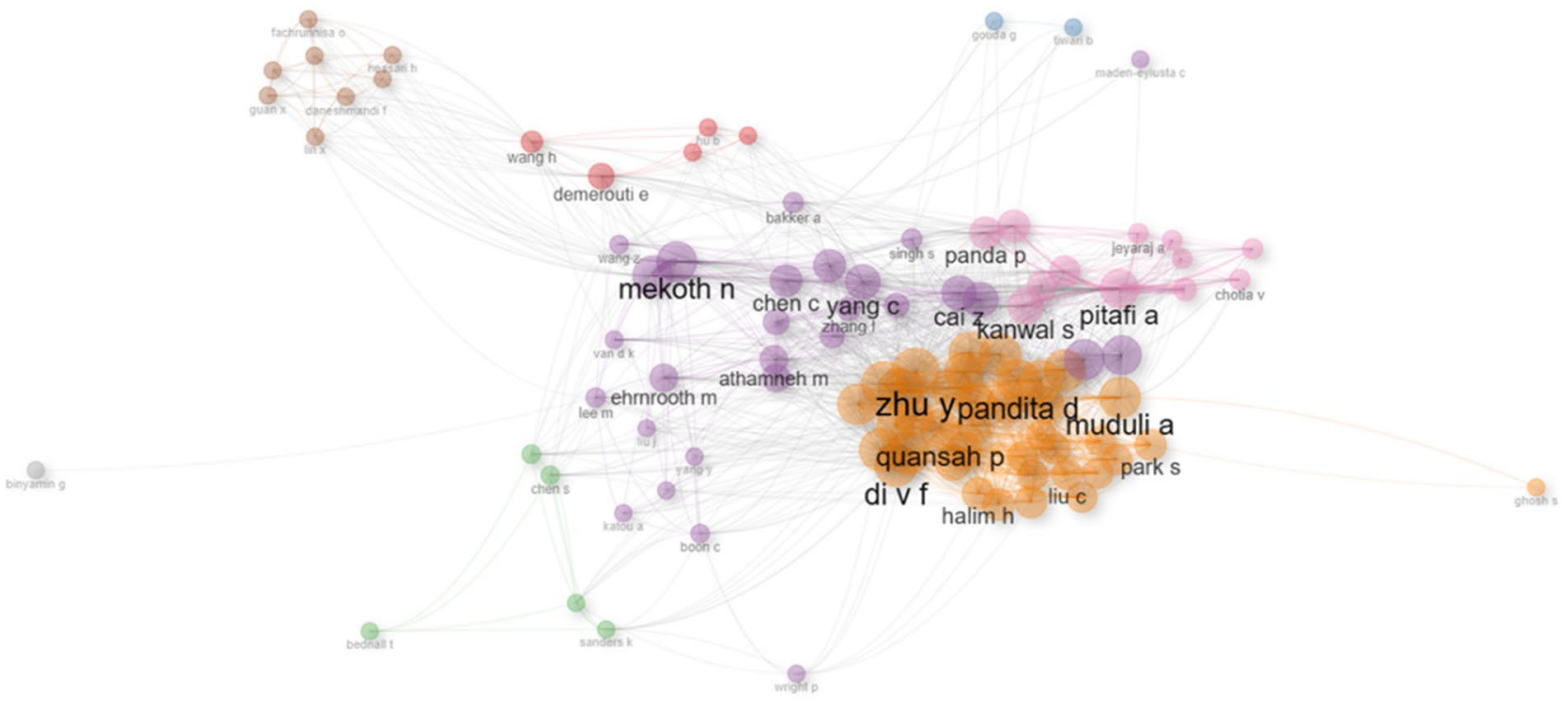
Co-citation network of employee agility articles’ authors (2000 – 2025). This figure shows groups of authors who are often cited together from the year 2000 to 2025.

A co-citation network analysis was performed to identify the intellectual structure in employee agility research, which comprises groups of authors who are often cited together. This analysis was based on descriptive data processed using Biblioshiny, and the results are shown in Figure 9. Figure 9 shows that over the past 25 years, the network of authors in the employee agility literature can be grouped into three main clusters. The first cluster, marked by orange nodes and networks and populated by authors such as Ashutosh Muduli, Deepika Pandita, Y. Zhu, and Pandya, represents psychological and individual-level approaches to employee agility. In this cluster, agility is understood as an adaptive capability related to employee psychological empowerment (Muduli and Pandya, 2018), an individual’s ability to respond to changes triggered by digital transformation and technological developments (Muduli and Choudhury, 2025; Rastogi and Pandita, 2025; Vapiwala et al., 2025), and its relationship to learning processes (Ghosh et al., 2021; Ghosh and Muduli, 2021; Srigouri and Muduli, 2024b). This cluster structure shows that employee agility is explained through psychological and learning mechanisms as the main micro-foundations at the individual level.

The second cluster, represented by purple nodes and networks and including authors such as Nandakumar Mekoth and MHA Athamneh, focuses on human resource agility and how organizational context influences employee agility. Unlike the HRM approach, which highlights specific managerial practices and systems, this cluster views agility as a human resource capability at the organizational or collective level, shaped by organizational support, structure, and readiness (Athamneh and Jais, 2023; Husni et al., 2023; Sony and Mekoth, 2017a, 2017b). Therefore, employee agility is understood as the outcome of interactions between the individual and the wider organizational environment. This cluster demonstrates a meso-level perspective that connects the structural view of HRM with the individual psychological approach.

The third cluster, led by authors such as Abdul Hameed Pitafi and Prerna Panda, shows a strong connection between individual employee agility and technological and knowledge-based processes. In this cluster, employee agility is understood as an adaptive capacity embedded in technology use and knowledge-sharing mechanisms (Lai et al., 2021; Pitafi, 2025; Pitafi et al., 2025a; Rasheed and Pitafi, 2025), as well as in organizational factors such as organizational virtuousness (Panda and Singh, 2025a, 2025b, 2026). The structure of this cluster reveals that employee agility is not just a result of technological or organizational influences but also relates to aspects of wellbeing and work behavior, such as job crafting. Therefore, this cluster highlights an approach that considers employee agility as an integrated capability within a dynamic socio-technological and organizational system.

Overall, the cluster structure resulting from the co-citation analysis shows that employee agility research has evolved through multiple complementary intellectual pathways. Psychological approaches at the individual level highlight the importance of internal readiness and learning processes as micro-foundations of agility, while meso-level approaches place employee agility within the context of human resource capabilities and collective organizational influence. Conversely, socio-technological approaches expand the understanding of employee agility as an adaptive capability embedded in a technology- and knowledge-driven work ecosystem. These connections between clusters demonstrate that employee agility does not develop in a straight line within a single perspective but is instead shaped by the interaction of individual, organizational, and contextual factors. Therefore, the results of this co-citation analysis confirm that employee agility is a multidimensional construct shaped by contributions from various intellectual traditions, thereby providing the conceptual foundation for structural and thematic mapping in future analyses.

#### 4.2.2 Bibliographic coupling

**Figure 10:**
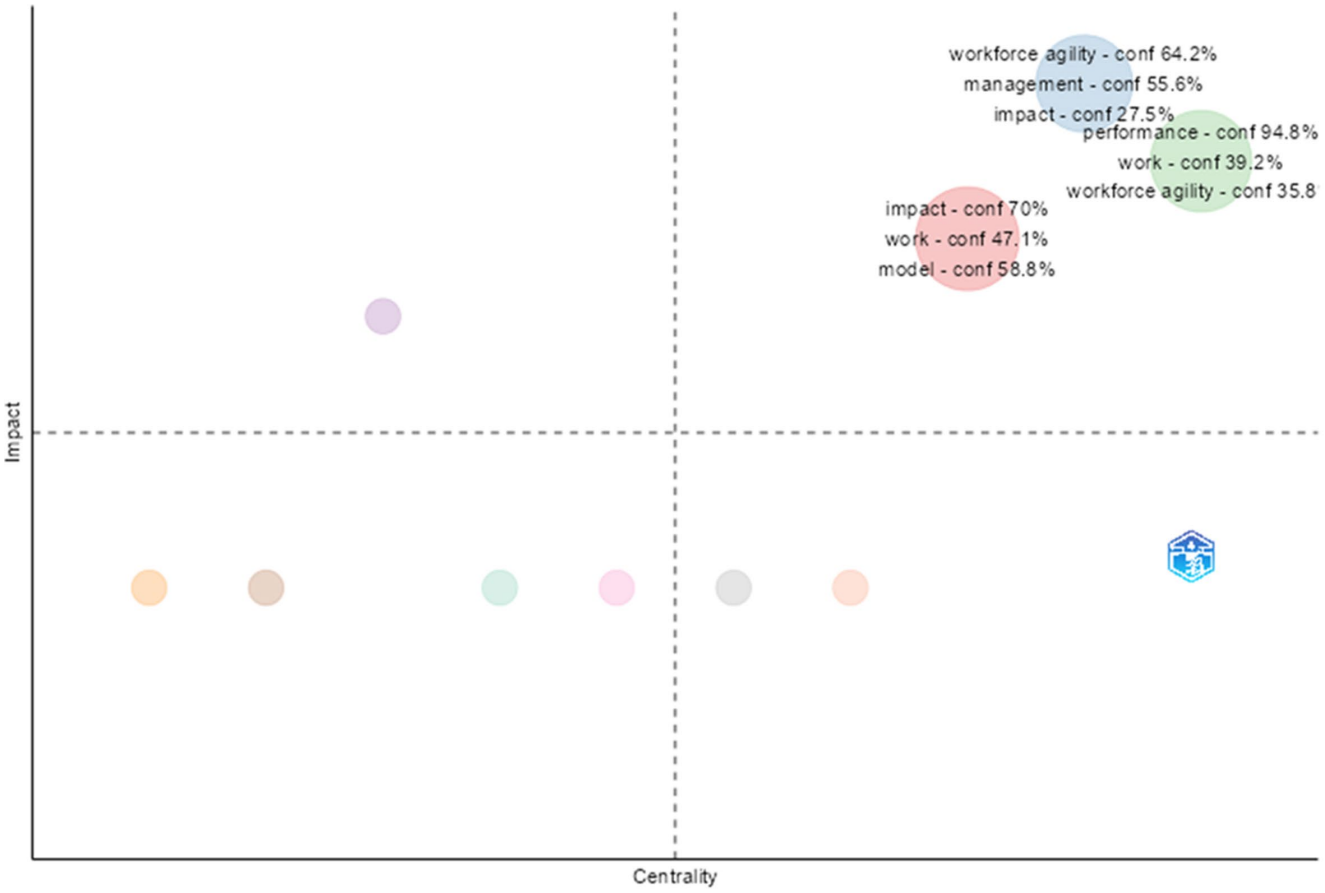
Bibliographic coupling of employee agility articles. This figure displays a theme is connection among themes in employee agility research from the years 2000 to 2025.

An analysis of the intellectual framework of employee agility research was also performed using bibliographic coupling to visualize the relationships between recent documents based on shared references. This illustrates the direction of current theoretical progress in employee agility studies. The results of the bibliographic coupling are presented in Figure 10, which maps research themes according to their centrality and impact. Centrality shows how connected a theme is to others in the research network, reflecting its importance or key position within the overall field. Impact, on the other hand, measures the maturity and internal strength of a theme, indicated by the number of supporting studies and the consistency of conceptual and methodological development. Combining these two measures allows themes to be classified into four groups: motor themes, niche themes, basic themes, and emerging or declining themes.

The analysis shows that workforce agility is a key and influential theme. This finding indicates that the current literature on employee agility is increasingly focusing on team-level agility and positioning workforce agility as an important concept that links different research areas. The role of workforce agility as a main theme marks a shift from viewing agility as an individual outcome to understanding it as a coordinated ability built into teamwork and organizational systems. Along with these primary themes, the bibliographic coupling map also highlights niche themes and those in emerging or declining fields. Although these themes have fewer connections to others, they show the diversification and expansion of employee agility research.

Specifically, the appearance of themes related to digitalization and individual adaptability in the quadrant shows that research on employee agility is increasingly focusing on integrating socio-technical factors and personal adaptive skills. Overall, this pattern indicates that while workforce agility remains a key focus in current research, studies on employee agility continue to evolve by combining individual and digital perspectives, which may shape future research directions. Therefore, this bibliographic coupling structure confirms the ongoing development of employee agility research, balancing both conceptual stability and thematic innovation, serving as a foundation for more detailed conceptual mapping in future studies.

#### 4.2.3 Co-word analysis

Whereas co-citation analysis maps the network of authors in the employee agility field, co-word analysis offers insight into how key concepts in the employee agility literature are interrelated and have evolved by analyzing the keywords appearing in that literature. The co-word analysis in this study draws on thematic maps produced by Biblioshiny and VOSviewer, as well as trending topic data from Biblioshiny. Before conducting the co-word analysis, a process of cleaning and grouping keywords was performed to ensure the results remained consistent and clear. The initial keywords were taken from the author keywords and index keywords of the included journal articles. First, an automated cleaning step was carried out using bibliometric software, which involved converting all terms to lowercase, removing duplicates, and standardizing singular and plural forms (e.g., “employee agility” and “employees’ agility”). This manual step followed established bibliometric practices to reduce fragmentation caused by different terminology. The final cleaned and grouped set of keywords was then used for the co-word analysis.

##### 4.2.3.1 Thematic map

**Figure 11:**
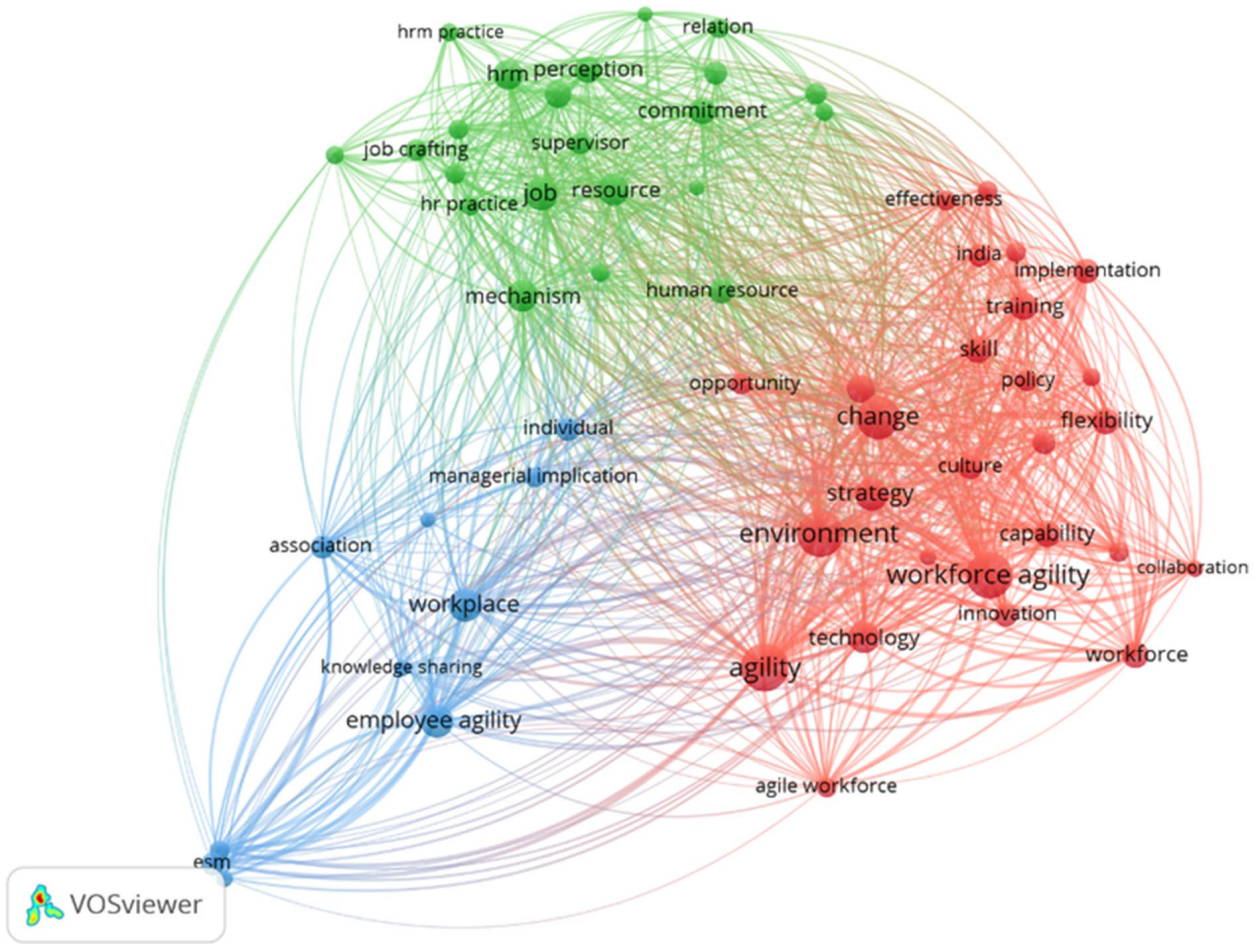
Thematic map of employee agility articles (Years 2000 – 2025). Analysed by VOSViewer. This figure shows main clusters of employee agility research.

One analysis performed in this study is a thematic map analysis based on the VOSviewer visualization of the dataset, as shown in Figure 11. The co-word network mapping results reveal three main clusters that shape the discourse of agility research. The red cluster centers on themes such as workforce agility, strategy, change, innovation, and technology, representing a research focus at both the organizational and macro-strategic levels. The authors and articles, such as Ciro Troise, Janani, Ciara Franco, and Petermann, are included in this cluster. This affirms that the literatures in this cluster suggests that employee agility emerges as a dynamic capability resulting from organizational efforts to cultivate employee intelligence and a collaborative culture, enabled by well-aligned and appropriately implemented policies (Franco and Landini, 2022; Petermann and Zacher, 2020; Troise et al., 2022). Apart from being positioned as an outcome, the literature in this cluster also frames employee agility as a central driver of innovation, sustainability, and successful change initiatives. Within the dynamic capabilities and digital transformation framework, an agile workforce constitutes a strategic capability that bridges turbulent environments and organizational performance, thereby supporting competitive advantage (Alviani et al., 2024; Janani and Vijayalakshmi, 2023, 2025; Petermann and Zacher, 2020, 2021).

Meanwhile, the green cluster consists of authors such as Athamneh, Prerna Panda, Nandakumar Mekoth, and Michael Sony, emphasizing that this cluster positions employee agility as the resuls of factors related to human resource management (HRM). This cluster highlights the important role of HR practices and individual mechanisms, including job crafting, supervisor support, commitment, and resources in shaping employee agility (Athamneh and Jais, 2023; Panda and Singh, 2026; Sony and Mekoth, 2016a, 2017b). Lastly, the blue cluster contains some authors, namely Abdul Hameed Pitafi and Muduli, so that some keywords such as workplace, knowledge sharing, and enterprise social media (ESM) are strongly linked in this cluster. This cluster illustrates how individual agility in the workplace can be nurtured through interaction, collaboration, and communication technology, which contributes to the formation of employee agility by gaining employees’ capabilities and psychological safety (Pitafi et al., 2019; Pitafi et al., 2025a; Zhu et al., 2023).

Overall, the three clusters reveal a layered yet fragmented body of agility research. The red cluster mainly reflects a strategic perspective, where agility is discussed in relation to innovation, organizational change, and performance in turbulent environments. In contrast, the green cluster shifts focus toward the managerial and HRM domains, highlighting how such capabilities are developed at the individual level. Meanwhile, the blue cluster emphasizes the everyday work context, stressing the importance of social interaction, collaboration, and digital communication in fostering adaptive behaviors. Although these perspectives are conceptually linked, they are often studied separately, with little effort to connect insights across different levels of analysis. Consequently, while the literature identifies multiple pathways for developing agility, it still falls short of explaining how individual and workplace processes are systematically linked to broader strategic and organizational outcomes.

**Figure 12:**
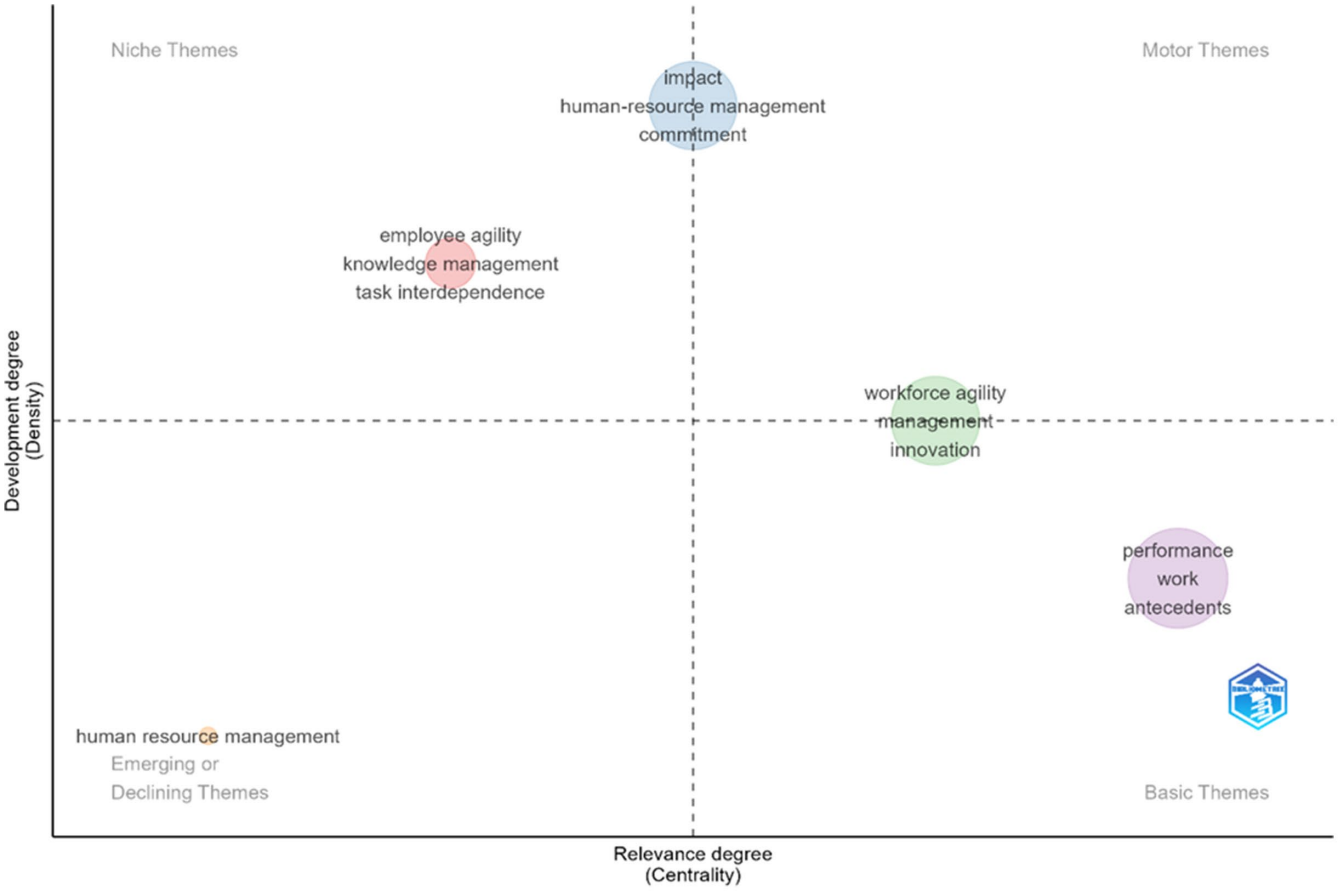
Thematic map based on bibliometrix. This figure shows the density and centrality of employee agility research.

These findings are also supported by a thematic map analysis using the Bibliometrix application, as shown in Figure 12. The thematic mapping illustrates how research on employee agility is distributed across four quadrants based on density and centrality. First, in the niche themes quadrant (high density—low centrality), topics such as *impact*, *human resource management*, and *commitment* appear. These themes have strong internal cohesion—meaning the existing studies on them are relatively solid and focused—but their contribution to the broader conceptual landscape remains limited. Thus, while important within their specific domain, these themes are not yet fully integrated into the main discourse on agility. Second, motor themes (high density—high centrality) have not yet shown a clearly dominant topic in this analysis. The absence of a strong motor theme suggests that agility research is still in a phase of theoretical consolidation and may require new driving themes to unify the field’s direction. Third, in the basic theme quadrant (low density—high centrality), we find topics like *performance*, *work*, and *antecedents*. These themes appear frequently and serve as conceptual foundations in the literature, particularly concerning the relationship between agility and performance. However, because their density is low (i.e., these studies do not form a very cohesive cluster), the work on these themes has been largely descriptive and has not yet developed deeper theoretical mechanisms. Fourth, emerging or declining themes (low density—low centrality) are represented by *human resource management*. This position suggests that although HRM was once a key reference in agility studies, its relevance in the current context appears to be waning or being supplanted by other, more contemporary perspectives.

Outside the four quadrants, there are transitional themes that stand out, such as *employee agility*, *knowledge management*, *task interdependence*, as well as *workforce agility*, *management*, and *innovation*. Their relatively central positions indicate that these topics are currently developing and have the potential to become motor themes in the future. In particular, the linkage between employee agility and innovation suggests a new direction in research, emphasizing agility not just as a response to change, but also as a driver for the creation of organizational innovation.

##### 4.2.3.2 Trending topics

**Figure 13:**
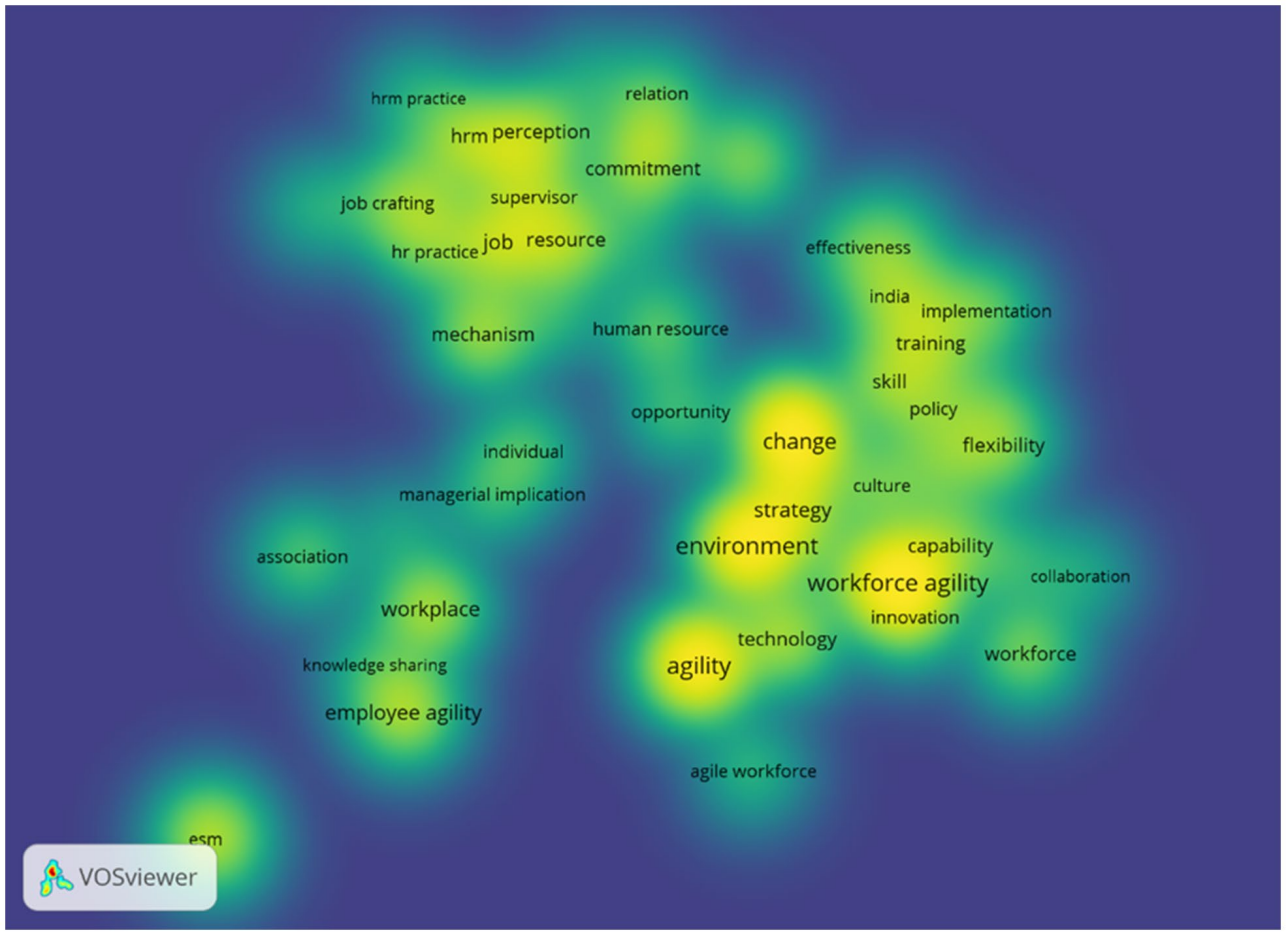
Density mapping of employee agility research topics. This figure shows themes density of employee agility research topics based on keywords from the years 2000 - 2025.

**Figure 14:**
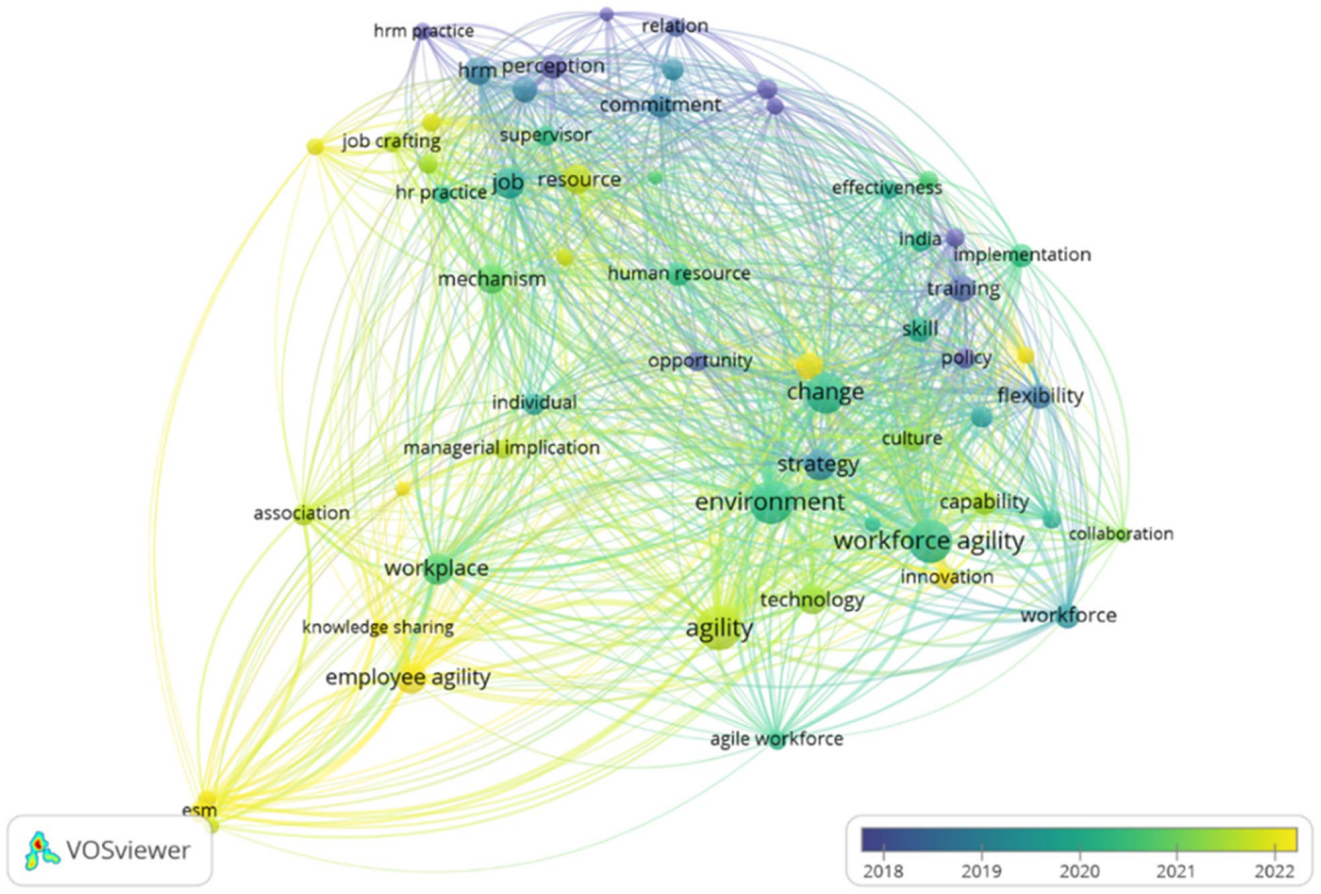
Overlay mapping of employee agility research topics. This figure shows a shift in the focus of employee agility research.

In addition to mapping the thematic structure of research, this analysis also highlights trending topics that have developed over time. This approach makes it possible to observe the temporal dynamics of employee agility research, identifying topics that are on the rise, relatively established themes, as well as issues that are beginning to lose relevance. Accordingly, the analysis of trending topics provides a clearer view of the research front and potential future research agendas. The trending topics analysis was performed using VOSviewer and Bibliometrix. The results from VOSviewer include density and overlay visualizations of employee agility research, as shown in Figures 13, 14. The overlay visualization (Figure 14) shows a shift in the focus of employee agility research from around 2018 to 2022. Early themes (indicated by bluish-purple colors) centered on human resource management practices, job resources, commitment, and relationships, reflecting an initial focus on foundational work behaviors and traditional HRM functions. Over time, research began to shift toward themes of organizational change and strategy, represented by keywords such as *change*, *environment*, *strategy*, and *workforce agility*. In the most recent period (indicated by yellow), research has increasingly emphasized issues such as *employee agility*, *knowledge sharing*, *training*, and *innovation*. This suggests that recent studies focus on the role of individuals and organizational mechanisms in addressing digital transformation and the increasingly complex dynamics of work environments.

Meanwhile, the density visualization (Figure 13) reinforces these findings by showing that the highest density areas cluster around keywords like *workforce agility*, *environment*, *strategy*, and *change*, indicating that these themes have been central to the discourse over the past two decades. However, there are other emerging “hotspots” starting to gain prominence, namely *employee agility* and *knowledge sharing*, although their intensity is still lower compared to the organization-level themes. This confirms that research at the individual level is growing but has not yet become dominant. In contrast, certain topics such as *enterprise social media (ESM)* and *association* appear more isolated with low density, indicating that those areas remain niche and present significant opportunities for future exploration.

**Figure 15:**
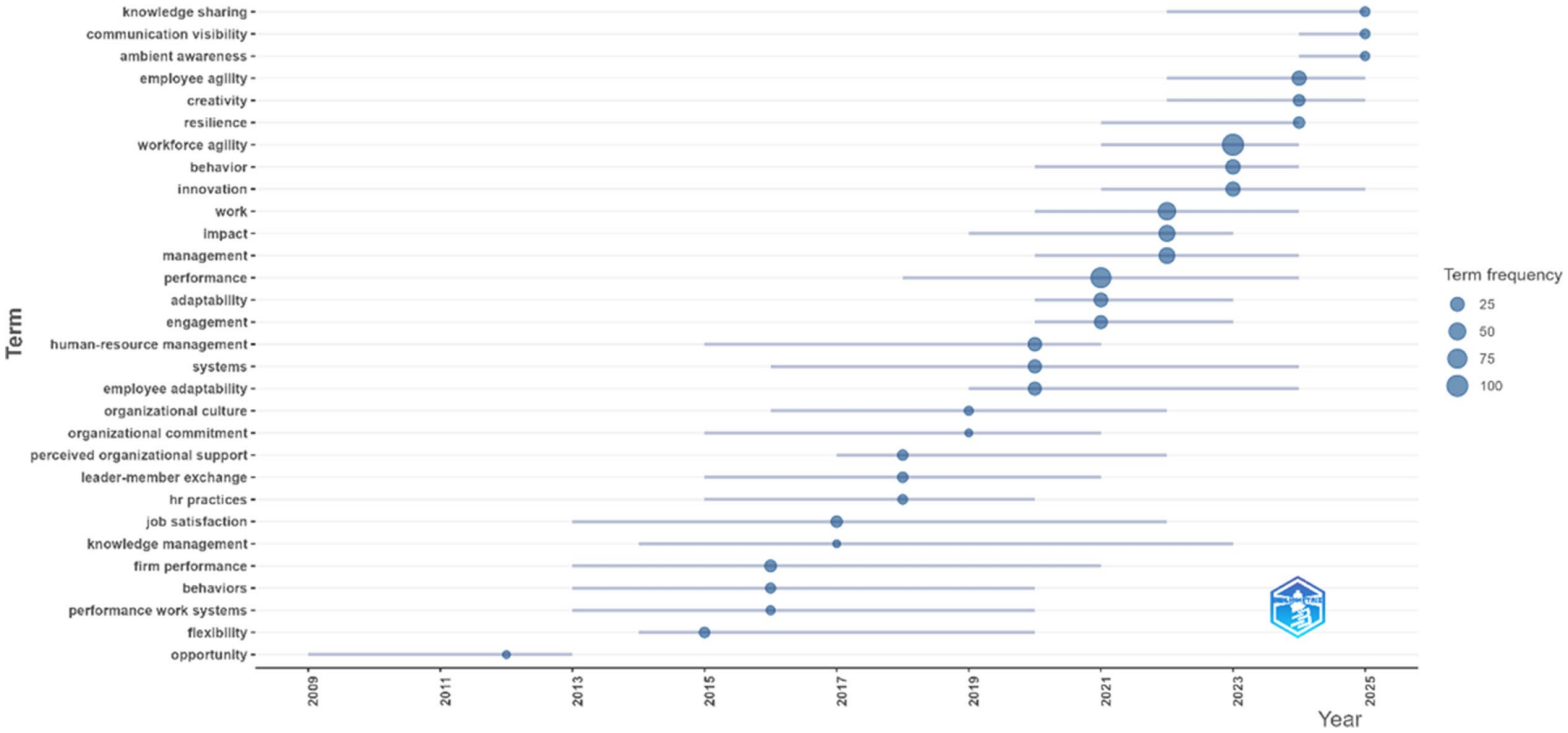
Trending topics based on bibliometrix analysis. This figure shows the terms used in employee agility research from the years 2000 - 2025.

Trending topics in employee agility field of research were also analyzed using Bibliometrix, with the results shown in Figure 15. The analysis of trending topics indicates that research on employee agility has undergone a significant shift in the last two decades. In the early phase, studies largely emphasized classic issues such as performance, management, and organizational culture, which provided the conceptual foundations for understanding agility in an organizational context. However, since 2018—particularly in the wake of the COVID-19 pandemic—there has been a sharp increase in new topics that are more contextual to digital transformation demands and disruptions in the work environment. Terms such as employee agility, workforce agility, adaptability, and impact have risen rapidly in frequency, indicating a growing academic interest in agility as a core capability of individuals and organizations.

### 4.3 Summary of findings

The performance analysis presented in Section 4.1 shows that research on employee agility has grown steadily and continues to accelerate, reflecting rising scholarly interest in how organizations build adaptive capacity in changing environments. At the same time, the science mapping results in Section 4.2 suggest that this growth has not followed a single, unified path. Rather than accumulating as a homogeneous body of knowledge, the field has evolved through several partially connected research streams. The co-citation patterns point to early intellectual roots that are closely tied to HRM and performance-oriented traditions, while the bibliographic coupling results indicate that these managerial perspectives continue to shape the field alongside emerging strategic and psychological approaches. At a more conceptual level, the co-word analysis shows that employee agility is increasingly discussed in strategic, technological, and knowledge-related contexts, indicating a gradual shift toward more capability-oriented and context-sensitive interpretations. Taken together, these findings suggest that the literature is expanding not only in volume but also in thematic richness, gradually moving beyond a predominantly HRM-centered framing toward a more multidimensional understanding that brings together psychological mechanisms, strategic responsiveness, and knowledge-driven organizational dynamics.

Overall, the results of the bibliometric analysis show that research on employee agility is developing through increasingly complex and multidimensional intellectual and conceptual dynamics. Co-citation analysis identifies several key intellectual traditions that form the basis for understanding employee agility, ranging from psychological approaches at the individual level, to meso-level perspectives emphasizing the role of organizational context and human resource agility, to socio-technological approaches linking agility to digital processes and knowledge-based interactions. Furthermore, bibliographic coupling analysis indicates that recent research is increasingly focusing on workforce agility as a collective capability, while also allowing for thematic differentiation that integrates digital aspects and individual adaptability. These findings are supported by thematic maps and trending topics analysis, which reveal a shift in research focus from traditional managerial and performance issues to viewing agility as a core capability related to innovation, learning, and organizational resilience amid changing work environments. Thus, these results confirm that employee agility is not a static, single construct, but rather a concept that continues to evolve through interactions across individual, organizational, and socio-technological levels, forming the foundation for the subsequent theoretical and conceptual discussion.

## 5 Discussion

Taken together, the performance and science mapping analysis suggest that research on employee agility is not only expanding in volume but also becoming increasingly differentiated in how the concept is understood. While early work remains closely tied to HRM and performance-focused traditions, more recent studies point to a gradual broadening of perspectives, with stronger links to strategic, psychological, and knowledge-based viewpoints. The co-word and coupling patterns indicate that agility is increasingly seen as a multidimensional capability shaped by strategic responsiveness, technological settings, and organizational context. At the same time, the visible separation among clusters suggests that this body of research has yet to reach full theoretical integration. Against this backdrop, the following section reflects on the implications of these developments for theory building, methodological approaches, and directions for future research.

### 5.1 The evolution and intellectual structure of employee agility research

Based on the findings from the publication performance analysis and knowledge mapping, research on employee agility shows a clear evolutionary pattern, both over time and conceptually. Regarding publication output, the slow growth of articles in the early stages, followed by a sharp increase since 2019, indicates that employee agility is becoming more recognized as a crucial factor in addressing the challenges of an uncertain work environment. This rise not only signifies increased research activity but also reflects a shift in academic focus toward agility as a strategic capability in response to the global crisis, rapid digital transformation, and post-pandemic work changes. As a result, employee agility has transitioned from a marginal topic in human resource management to a central theme in modern organizational and management discussions.

From an intellectual structure perspective, the results of science mapping show that the development of employee agility research is shaped by several complementary yet not fully integrated intellectual traditions. Co-citation analysis reveals a research cluster rooted in an individual psychological approach, emphasizing psychological conditions, learning, and empowerment as the foundation of agility. Conversely, another cluster reflects a meso-level approach that views agility as a collective human resource capability influenced by organizational support and context. Additionally, a socio-technological cluster has emerged, linking employee agility to digital technology, knowledge sharing, and platform-based interactions. The presence of these clusters confirms that employee agility does not develop along a single theoretical path but rather through interactions among different perspectives, highlighting the complexity of the phenomenon under study.

These findings are supported by bibliographic coupling and thematic mapping results, which highlight the dynamics of thematic consolidation and differentiation in current research. Workforce agility emerges as a central theme linking various contemporary research streams, showing a trend toward consolidation at the collective and organizational levels. At the same time, the thematic map and trending topics analysis reveal the emergence of new themes focused on employee agility, knowledge sharing, innovation, and resilience. This pattern reflects the literature’s efforts to expand the understanding of agility beyond simply an adaptive organizational response to a multidimensional capability rooted in the individual and continuous learning processes.

Overall, the performance analysis and science mapping results show that employee agility research is in an early stage of maturity, characterized by rapid publication growth and a variety of conceptual approaches. However, this maturity is still incomplete, as integration between the individual, organizational, and socio-technological contexts has not yet been fully achieved. Therefore, the development of employee agility research can be viewed as a process shifting from initial fragmentation to the need for a more integrated conceptual framework that can explain agility as an adaptive ability functioning across different levels and contexts.

### 5.2 The missing link and future direction of employee agility research

Based on the synthesis of publication performance analysis and intellectual structure mapping, research on employee agility is experiencing rapid growth but still has opportunities for conceptual and contextual improvements. Although the literature has expanded greatly over the past 20 years, with more publications, diverse themes, and the emergence of various interdisciplinary approaches, science mapping results show that connections between different levels of analysis and research traditions remain somewhat limited. Therefore, future research on employee agility should focus on bridging these gaps and enhancing understanding of agility as an adaptive capability that functions dynamically across various organizational settings. Some missing links and future directions of employee agility research are defined as follows.

#### 5.2.1 The integrated research framework

The results of the bibliometric analysis, both in performance analysis and in science mapping analysis, show that there remains a need for research that integrates agility at the individual (micro) level with agility at the work team (meso) and organizational (macro) levels. Previous literature has examined the role of organizations in shaping employee agility capabilities (Alavi et al., 2014; Breu et al., 2002; Franco and Landini, 2022; Harsch and Festing, 2020; Muduli, 2016, 2017; Sherehiy and Karwowski, 2014), the role of employee agility in translating the role of organizations in improving adaptive performance or performance in general during times of change (Alviani et al., 2024; Muduli, 2016; Salmen and Festing, 2022; Varshney and Varshney, 2024), and the role of agility as an antecedent of organizational agility and organizational performance (Alviani et al., 2024; Franco and Landini, 2022; Petermann and Zacher, 2022; Prieto and Talukder, 2023). However, although these studies have developed in parallel, those that explicitly position employee agility as a micro-foundation that connects and drives agility at the meso and macro levels remain relatively limited.

The literature on employee and workforce agility shows that most research is fragmented by level of analysis. At the individual level (micro-level), employee agility is generally understood as an individual’s capability to behave adaptively, proactively, and resiliently in the face of change (Alavi and Wahab, 2013; Alviani et al., 2024; Breu et al., 2002; Petermann and Zacher, 2022; Rasheed and Pitafi, 2025; Storme et al., 2020). Employee agility is also often associated with psychological factors and individual competencies, such as psychological empowerment (Cyfert et al., 2022; Muduli, 2017; Storme et al., 2020) and the use of digital technology (Durst et al., 2023; Ghosh et al., 2021; Muduli and Choudhury, 2025; Pitafi, 2025). At the organizational level (meso-level), agility is more often positioned as a result of managerial practices, organizational structure, human resource management, and flexible work systems (Alavi et al., 2014; Breu et al., 2002; Franco and Landini, 2022; Harsch and Festing, 2020; Muduli, 2016, 2017; Sherehiy and Karwowski, 2014), while at the institutional or environmental level (macro-level), employee agility is influenced by regulatory pressures, industry dynamics, and external environmental uncertainty (Ajgaonkar et al., 2022; Muduli, 2016; Salmen and Festing, 2022; Walter, 2021). Most empirical research still treats employee agility as a stand-alone independent or mediating variable, without explicitly linking it to the formation of organizational or institutional agility within a dynamic framework. Thus, although the literature is rich in identifying the antecedents and consequences of agility, the mechanisms by which employee agility contributes to the formation of cross-level agility through dynamic capability mechanisms have not yet been systematically formulated.

For example, a study by Petermann and Zacher (2022) highlighted the importance of the interaction of various factors at the individual, team, and organizational levels in fostering the formation of agile organizations. Meanwhile, research by Franco and Landini (2022), Lai et al. (2021), and Muduli (2017) demonstrated that individual technological and digital competencies can drive competency-based work structures, ultimately fostering organizational agility. Research that explicitly addresses the role of multilevel employee agility is conducted by Junker et al. (2022), who demonstrated that team-level agility can shape collective norms that enable individuals within the team to perform well. However, this research focused more on how meso-level agility shapes micro-level agility, while also examining how the collective influence of micro-level agility shapes meso- and macro-level agility.

The dynamic capabilities perspective highlights that organizational capabilities develop from the activities, behaviors, and interactions of actors at the micro level, which are then combined into capabilities at the meso and macro levels. In this view, employee agility can be seen as a micro-foundation that allows organizations to respond to change quickly and sustainably. Therefore, further research should create an integrated framework that explains these cross-level mechanisms and explores how to increase agility at each level by utilizing organizational resources, including the role of top management, a culture and climate that foster agility, and the dynamics of internal and external pressures.

#### 5.2.2 Research stream integration

The bibliometric analysis also shows that over the past 25 years, much of the literature has tended to associate employee agility with human resource management practices. The results of thematic mapping and trending topics analysis indicate a shift in research focus from traditional managerial practices to issues related to digitalization, innovation, and knowledge-based learning. However, the literature still reveals a tendency toward two fairly separate research streams: a human resource management-based approach and a digital technology-based approach. While employee agility is indeed closely related to HRM, environmental factors—especially digital transformation—cannot be ignored. One of the clearest gaps in the literature is the divide between one stream of research focusing on human resource-driven agility and another emphasizing digital-driven agility. Agility is seen as an enabler of innovation and change; therefore, innovation and digital competencies that help develop and strengthen agility are essential areas of research.

Traditional agility research (what we might call HR-driven agility) has largely highlighted the role of HRM practices, job commitment, employee engagement, and performance as primary antecedents of agility (Aldabbagh, 2023). This perspective frames agility as a product of effective human resource management practices—for example, through talent management, supportive leadership, and training programs (Jager et al., 2022). In this view, human resource-driven agility centers on an organization’s structural factors and human interactions as the foundation for developing individual agility.

In contrast, recent research trends increasingly emphasize digital-driven agility, namely the role of technology, digitalization, and digital competence as key determinants. Studies such as Alviani et al. (2024) and Pitafi et al. (2025a) highlight how the use of enterprise social media (ESM), AI-based systems, and knowledge-sharing platforms can drive individuals to become more adaptive, responsive, and innovative. In this context, agility is no longer seen merely as an outcome of traditional HR practices, but as arising from individuals’ ability to acquire digital competencies and interact productively within a digital ecosystem.

However, our bibliometric results show that these two streams of research have thus far progressed in parallel with little conceptual integration. Human resource-driven agility research is more prominent in the management and HR literature, whereas digital-driven agility appears frequently in the innovation, information systems, and technology management literature. Few studies explicitly bridge the two—for instance, by examining how HRM practices can enhance employees’ digital competencies, or how digital tools and analytics can strengthen the effectiveness of HR-driven agility initiatives. Yet, such integration is important for providing a comprehensive understanding of how employee agility is cultivated in the VUCA era.

This gap represents a significant opportunity for future research. Empirical studies can be directed toward testing multilevel models that integrate HRM factors with digitalization factors. For example, one could examine a model in which empowering leadership and talent management (as HR-driven antecedents) are combined with digital competence, AI adoption, and ESM usage (as digital-driven antecedents), to assess their joint impact on employee agility and organizational innovation (Naim et al., 2024; Zhang et al., 2022). Such integration would not only enrich theoretical understanding but also offer practical guidance for designing more comprehensive organizational strategies to develop agility. Therefore, future research needs to integrate these two perspectives, for example, by examining how HRM practices, leadership, and talent development can strengthen individual digital capabilities, and how digital technology can act as an enabler for the effectiveness of HRM practices in fostering employee agility.

#### 5.2.3 The limited exploration of certain dimensions and context

Another aspect that needs attention in shaping agile individuals is the role of leadership and the presence of employee trust in leadership (Zhang et al., 2022). During times of organizational change, leaders who can maintain operational stability while empowering organizational resources and encouraging innovation are key to fostering agility. Prior research has shown that transformational leadership can enhance employee performance by developing individual potential and articulating a long-term organizational vision (Ali et al., 2025; Islam et al., 2021, 2022; Lee et al., 2023). Leadership behaviors that are oriented toward employees as valuable resources can influence employees’ feelings about change, their trust and understanding of changes taking place, as well as their performance and resistance to those changes (Weber et al., 2022). Employee trust in leadership is particularly critical during periods of change; thus, leadership styles that are transformational, servant-oriented, foster collaboration, and use reward systems in performance management should be employed to encourage employee behaviors that contribute to organizational agility (Musaigwa, 2023). Leaders must also be cautious in applying dual or multiple leadership styles, since not all combinations of styles may benefit the organization (Weber et al., 2022). The importance of building employee trust in leadership during times of change has not yet been supported by sufficient empirical research, especially concerning the relationship between trust in leadership and the development of employee agility that leads to optimal performance.

Moreover, in light of organizations’ need to maintain good performance and ensure sustainability in volatile environments, ambidextrous leadership—a leadership style that flexibly balances exploration and exploitation—has been suggested as well-suited to organizational needs (Khan et al., 2025; Zhang and Suntrayuth, 2024). Ambidextrous leadership has been found to have a positive and significant effect on agility by increasing an organization’s ability to respond to change while simultaneously innovating (Zhang and Suntrayuth, 2024). It has also been empirically shown to foster innovative work behaviors, increase work engagement, and build the talent agility required to develop an agile organization (Gouda and Tiwari, 2024). Other findings suggest that the combination of ambidextrous leadership and organizational agility drives sustainable innovation and business transformation (Khan et al., 2025). However, there is still a lack of research explicitly examining the role of ambidextrous leadership in fostering employee agility.

The analysis also highlights the limited exploration of psychological and sustainability dimensions in employee agility research. Although factors such as empowerment, mindset, and wellbeing have begun to receive attention in recent studies, they have not yet been fully integrated into the core framework of agility research. Future research could broaden its focus by viewing employee agility not only as a short-term adaptation mechanism but also as a capability that supports the long-term sustainability of individuals and organizations. This approach would enable a more comprehensive understanding of the relationship between agility, psychological health, and organizational resilience. Future research should also pay greater attention to psychological dimensions and sustainability factors related to employee agility. Aspects such as employee wellbeing, career adaptability, and psychological empowerment can function not only as antecedents of agility, but also as important outcomes that support individual resilience and organizational sustainability (Storme et al., 2020). By treating psychological factors as integral to employee agility, future studies can broaden understanding to recognize that agility is not just a short-term adaptive capability but also a strategic capability for ensuring long-term sustainability in a VUCA world.

The bibliometric analysis also reveals critical limitations in the contexts of existing research on employee agility. Highly cited publications are still dominated by authors and institutions from the United States, India, and China, whereas research from Southeast Asia remains very limited. The dominance of these three countries in the agility literature can be understood, given their well-established research ecosystems and the intense digital transformation dynamics they face. However, investigating employee agility in Southeast Asian contexts is important because countries in this region have distinctive bureaucratic structures, work cultures, leadership styles, and societal conditions, which may differ from Western contexts. The applicability of agility-related theories and practices needs to be tested under these differing conditions to ensure their relevance and effectiveness.

Another context limitation is the heavy focus on private or commercial sector organizations, with far fewer studies conducted in government or public sector contexts. This gap is noteworthy because government organizations often have bureaucratic characteristics and face constraints such as limited resources and complex regulations, which can engender resistance to change. Building an agile government could foster innovation, continuous learning, and cross-unit collaboration, thus improving the performance of public institutions by enhancing collaboration and increasing the capacity to address complex social problems (Neumann et al., 2024).

In the face of a growing need for government organizations to also become agile—so that they can be responsive and adaptive to change—empirical research measuring how agility is formed, especially at the micro (individual) level in the public sector, is still scarce (Fischer and Neumann, 2024; Kühler et al., 2024; Neumann et al., 2024; Neumann et al., 2024). Neumann et al. (2024) suggest that adopting agility principles in government organizations should prioritize developing agility internally first. They describe forms of agility in government entities as “agile as a culture change,” “agile as governance,” and “agile as methodology.” Fischer and Neumann (2024) further argue that agility in government can be applied at three levels: macro (institutional), meso (organizational), and micro (individual). At the macro level, agility involves responses to external environmental changes, bureaucratic reforms, and regulatory adjustments; at the meso level, agility involves organizational restructuring, process redesign, and project management changes; and at the micro level, agility is reflected in individuals or teams through adaptive attitudes and behaviors. Therefore, empirical studies that examine the antecedents or impacts of employee agility at the micro level in government organizations are needed.

## 6 Conclusion

This study offers a comprehensive overview of how employee agility research has developed over the past 25 years, using a bibliometric approach that combines publication performance analysis with intellectual structure mapping. The findings indicate a clear shift in how employee agility has been understood over time. Originally rooted in human resource management practices and performance-focused perspectives, the concept has gradually evolved into a more multidimensional view of agility as an adaptive capability that is increasingly vital in environments marked by workplace uncertainty, rapid digital transformation, and changing work practices following the COVID-19 pandemic. The surge in recent publications reflects heightened academic interest in the strategic importance of agility, not only at organizational and team levels but also among individual employees. The science mapping results further show that employee agility research has developed through several interconnected yet partly distinct intellectual traditions. These include psychological approaches emphasizing individual adaptation, meso-level perspectives focusing on organizational context and managerial practices, and socio-technological approaches linking agility to digitalization and knowledge sharing. Together, these streams suggest that employee agility cannot be understood through a single theoretical lens. Instead, it emerges as a capability shaped by the interaction of individual traits, organizational structures, and broader contextual factors. Overall, the findings show that while the field of employee agility is approaching an early stage of quantitative maturity, significant opportunities still exist for deeper conceptual integration across levels of analysis, theoretical perspectives, and empirical contexts. In this way, the study not only traces the intellectual development of employee agility research but also lays a conceptual foundation for future work aimed at developing more integrated and contextually grounded theories and empirical studies.

This study makes several important contributions to the employee agility literature. First, it enhances understanding of how the concept has evolved by documenting a shift from a narrow focus on HRM practices toward a broader view of agility as a multidimensional, cross-level adaptive capability. By integrating insights from co-citation analysis, bibliographic coupling, and thematic mapping, the study shows that employee agility is shaped by the interaction among psychological, organizational, and socio-technological perspectives, which have often been examined separately. Second, the findings highlight employee agility as a potential microfoundation for workforce and organizational agility. By emphasizing the connection between individual adaptive behaviors and collective organizational capabilities, this study extends existing agility research and paves the way for multilevel theoretical frameworks that better explain how organizations build and sustain adaptive capacity. Third, this study contributes to a conceptual reorientation of employee agility, moving beyond its treatment as a reactive response to change. Instead, agility is increasingly understood as a proactive capability that supports innovation, continuous learning, and organizational resilience. This shift broadens the theoretical role of employee agility from a short-term survival mechanism to a key driver of long-term value creation. Methodologically, this study demonstrates the value of adopting an integrative bibliometric approach. By combining publication performance analysis with multiple science mapping techniques, the study not only maps the structural landscape of employee agility research but also captures its conceptual evolution over time. The use of a comprehensive dataset spanning 25 years enables the identification of both stable intellectual foundations and emerging research frontiers. More broadly, the findings illustrate how bibliometric analysis can be used reflexively to support theory development and synthesize complex bodies of knowledge, even without additional qualitative methods.

Despite its contributions, this study has several limitations. First, the analysis is restricted to English-language journal articles indexed in a single database, so relevant studies published in other languages or indexed elsewhere may not be fully included. Second, the bibliometric approach depends on citation relationships and keyword frequencies and therefore cannot replace in-depth qualitative analysis of individual articles. As a result, the conceptual interpretations here reflect broad patterns in the literature rather than detailed theoretical comparisons. Third, while this study identifies trends and conceptual gaps, it does not aim to establish causal relationships among variables. Future empirical research is therefore necessary to test and refine the conceptual insights presented here across various organizational settings, sectors, and institutional contexts.

## References

[ref1] Ajgaonkar S. Neelam N. G. Wiemann J. (2022). Drivers of workforce agility: a dynamic capability perspective. Int. J. Organ. Anal. 30, 951–982. doi: 10.1108/IJOA-11-2020-2507

[ref2] Alavi S. Abd Wahab D. Muhamad N. Arbab Shirani B. (2014). Organic structure and organisational learning as the main antecedents of workforce agility. Int. J. Prod. Res. 52, 6273–6295. doi: 10.1080/00207543.2014.919420

[ref9001] Alavi S. Wahab D. A. (2013). A review on workforce agility. Res. J. Appl. Sci 5, 4195–4199. doi: 10.19026/rjaset.5.4647

[ref3] Albrecht V. (2024). Collaborative competences for agile public servants: a case study on public sector innovation fellowships. Inf. Polity 29, 217–234. doi: 10.3233/ip-230056

[ref4] Aldabbagh T. (2023). The role of talent management in promoting organizational agility empirical study from the governmental sector in the United Arab Emirates: a conceptual framework. JPAIR Multidisciplinary Res. 52, 13–26. doi: 10.7719/jpair.v52i1.618

[ref5] Ali I. Gligor D. Balta M. Papadopoulos T. (2025). Leadership style’s role in fostering supply chain agility amid geopolitical shocks. Ind. Mark. Manag. 124, 212–223. doi: 10.1016/j.indmarman.2024.11.015

[ref6] Alviani D. Hilmiana W. S. Muizu W. O. Z. (2024). Workforce agility: a systematic literature review and research agenda. Front. Psychol. 15. doi: 10.3389/fpsyg.2024.1376399 PMC11422224 39323587

[ref7] Atanassova I. Bednar P. Khan H. Khan Z. (2025). Managing the VUCA environment: the dynamic role of organizational learning and strategic agility in B2B versus B2C firms. Ind. Mark. Manag. 125, 12–28. doi: 10.1016/j.indmarman.2024.12.008

[ref8] Athamneh M. H. A. Jais J. (2023). Analyzing the influence of decision making and effective employee communication on human resource agility in commercial banks: a mediation role for job satisfaction analyzing the influence of decision making and effective employee communication on human. Cogent Social Sciences 9:989. doi: 10.1080/23311886.2023.2203989

[ref9] Beltrán-Martín I. Roca-Puig V. Escrig-Tena A. Bou-Llusar J. C. (2008). Human resource flexibility as a mediating variable between high performance work systems and performance. J. Manage. 34, 1009–1044. doi: 10.1177/0149206308318616

[ref9003] Breu K. Hemingway C. J. Strathern M. Bridger D. (2002). Workforce agility: the new employee strategy for the knowledge economy. J. Inf. Technol. 17, 21–31. doi: 10.1080/02683960110132070

[ref10] Bukar U. A. Sayeed M. S. Razak S. F. A. Yogarayan S. Amodu O. A. Mahmood R. A. R. (2023). A method for analyzing text using VOSviewer. MethodsX 11:102339. doi: 10.1016/j.mex.2023.102339, 37693657 PMC10491643

[ref12] Chebat J.-C. Kollias P. (2000). The impact of empowerment on customer contact employees’ roles in service organizations. J. Serv. Res. 3, 66–81. doi: 10.1177/109467050031005

[ref13] Chen X. Chen J. Wu D. Xie Y. Li J. (2016). Mapping the research trends by co-word analysis based on keywords from funded project. Procedia Comput. Sci. 91, 547–555. doi: 10.1016/j.procs.2016.07.140

[ref14] Chong Y. K. Zainal S. R. M. (2024). Employee agility’s moderating role on the link between employee vitality, digital literacy and transformational leadership with job performance: an empirical study. Cogent Bus. Manag. 11:447. doi: 10.1080/23311975.2024.2337447

[ref15] Christofi M. Pereira V. Vrontis D. Tarba S. Thrassou A. (2021). Agility and flexibility in international business research: a comprehensive review and future research directions. J. World Bus. 56:101194. doi: 10.1016/j.jwb.2021.101194

[ref16] Cullen K. L. Edwards B. D. Casper W. C. Gue K. R. (2014). Employees’ adaptability and perceptions of change-related uncertainty: implications for perceived organizational support, job satisfaction, and performance. J. Bus. Psychol. 29, 269–280. doi: 10.1007/s10869-013-9312-y

[ref9004] Cyfert S. Szumowski W. Dyduch W. Zastempowski M. Chudziński P. (2022). The power of moving fast: responsible leadership, psychological empowerment and workforce agility in energy sector firms. Heliyon 8:e11188. doi: 10.1016/j.heliyon.2022.e11188 36311364 PMC9615033

[ref18] Donthu N. Kumar S. Mukherjee D. Pandey N. Lim W. M. (2021). How to conduct a bibliometric analysis: an overview and guidelines. J. Bus. Res. 133, 285–296. doi: 10.1016/j.jbusres.2021.04.070

[ref19] Dubey R. Bryde D. J. Dwivedi Y. K. Graham G. Foropon C. Papadopoulos T. (2023). International journal of production economics dynamic digital capabilities and supply chain resilience: the role of government effectiveness. Int. J. Prod. Econ. 258:108790. doi: 10.1016/j.ijpe.2023.108790

[ref9005] Durst S. Davila A. Foli S. Kraus S. Cheng C.-F. (2023). Antecedents of technological readiness in times of crises: a comparison between before and during COVID-19. Technol. Soc. 72. doi: 10.1016/j.techsoc.2022.102195

[ref20] Fischer C. Neumann O. (2024). Introduction to the special issue ‘towards a multi-level understanding of agile in government: macro, Meso and Micro perspectives.’. Inf. Polity 29, 123–136. doi: 10.3233/ip-249007

[ref21] Flavio J. Falla S. Karwowski W. (2024). Applied sciences survey-based studies of the agility construct in the healthcare sector: a systematic literature review. Appl. Sci. 1–26. Available online at: https://www.mdpi.com/2076-3417/14/3/1097

[ref22] Franco C. Landini F. (2022). Organizational drivers of innovation: the role of workforce agility. Res. Policy 51:104423. doi: 10.1016/j.respol.2021.104423

[ref23] Ghosh S. Muduli A. (2021). Learning agility, culture and outcome: an empirical study. Int. J. Indian Cult. Bus. Manag. 23, 95–110. doi: 10.1504/IJICBM.2021.115413

[ref24] Ghosh S. Muduli A. Pingle S. (2021). Role of e-learning technology and culture on learning agility: an empirical evidence. Hum. Syst. Manag. 40, 235–248. doi: 10.3233/HSM-201028

[ref26] Gouda G. K. Tiwari B. (2024). Ambidextrous leadership: a distinct pathway to build talent agility and engagement. Hum. Resour. Dev. Int. 27, 133–141. doi: 10.1080/13678868.2022.2163101

[ref9006] Harsch K. Festing M. (2020). Dynamic talent management capabilities and organizational agility-a qualitative exploration. Hum. Resour. Manag. 59, 43–61. doi: 10.1002/hrm.21972

[ref27] Hopp W. J. Tekin E. Van Oyen M. P. (2004). Benefits of skill chaining in serial production lines with cross-trained workers. Manag. Sci. 50, 83–98. doi: 10.1287/mnsc.1030.0166

[ref28] Husni M. Athamneh A. Jais J. (2023). Factors affecting human resource agility: a literature review and future research directions factors affecting human resource agility: a literature review and future research directions. Cogent Bus. Manag. 10:181. doi: 10.1080/23311975.2023.2193181

[ref29] Islam M. N. Furuoka F. Idris A. (2021). Mapping the relationship between transformational leadership, trust in leadership and employee championing behavior during organizational change. Asia Pac. Manag. Rev. 26, 95–102. doi: 10.1016/j.apmrv.2020.09.002

[ref30] Islam M. N. Furuoka F. Idris A. (2022). Transformational leadership and employee championing behavior during organizational change: the mediating effect of work engagement. South Asian J. Bus. Stud. 11, 1–19. doi: 10.1108/SAJBS-01-2020-0016

[ref31] Jafari-sadeghi V. Amoozad H. Busso D. Yahiaoui D. (2022). Technological forecasting and social change towards agility in international high-tech SMEs: exploring key drivers and main outcomes of dynamic capabilities. Technol. Forecast. Soc. Change 174:121272. doi: 10.1016/j.techfore.2021.121272

[ref17] Jager S. B. D. Born M. P. van der Molen H. T. (2022). The relationship between organizational trust, resistance to change and adaptive and proactive employees’ agility in an unplanned and planned change context. Appl. Psychol. 71, 436–460. doi: 10.1111/apps.12327

[ref32] Janani M. Vijayalakshmi V. (2023). An arts-based process to build workforce agility. J. Organ. Change Manag. 36, 917–931. doi: 10.1108/JOCM-03-2023-0092

[ref33] Janani M. Vijayalakshmi V. (2025). Agility and the three musketeers: the interplay of epistemic curiosity, joy, and rumination. Acta Psychol. 258:105215. doi: 10.1016/j.actpsy.2025.105215, 40580918

[ref34] Jindal H. Kumar D. Kumar S. Kumar R. (2021). Role of artificial intelligence in distinct sector: a study. Asian J. Comput. Sci. Technol. 10, 18–28. doi: 10.51983/ajcst-2021.10.1.2696

[ref35] Jooss S. Collings D. G. Mcmackin J. Dickmann M. (2024). A skills-matching perspective on talent management: developing strategic agility. Hum. Res. Manage. 63, 141–157. doi: 10.1002/hrm.22192

[ref9008] Junker T. L. Bakker A. B. Gorgievski M. J. Derks D. (2022). Agile work practices and employee proactivity: a multilevel study. Hum. Relat. 75, 2189–2217. doi: 10.1177/00187267211030101

[ref36] Kehoe R. R. Wright P. M. (2013). The impact of high-performance human resource practices on employees’ attitudes and behaviors. J. Manage. 39, 366–391. doi: 10.1177/0149206310365901

[ref37] Khan A. Khatoon H. Akram A. (2025). Impact of ambidextrous leadership style on project success: understanding the role of innovative work behaviour and workforce agility in the software industry. Pakistan Soc. Sci. Rev. 9, 29–38. doi: 10.35484/pssr.2025(9-II)03

[ref38] Kühler J. Drathschmidt N. Großmann D. (2024). ‘Modern talking’: narratives of agile by German public sector employees. Inf. Polity 29, 199–216. doi: 10.3233/ip-230059

[ref39] Lai H. Pitafi A. H. Hasany N. Islam T. (2021). Enhancing employee agility through information technology competency: an empirical study of China. SAGE Open 11:6687. doi: 10.1177/21582440211006687

[ref40] Lee C. C. Yeh W. C. Yu Z. Lin X. C. (2023). The relationships between leader emotional intelligence, transformational leadership, and transactional leadership and job performance: a mediator model of trust. Heliyon 9:e18007. doi: 10.1016/j.heliyon.2023.e18007, 37534000 PMC10391948

[ref42] Lin C. P. Hu S. W. Chiu C. K. (2025). Achieving job performance through agility and innovativeness by strategizing learning ambidexterity. J. Manage. Psychol. 40:752. doi: 10.1108/JMP-12-2023-0752

[ref43] Liu J. Soomro H. J. (2025). Technology meets psychology: digital finance integration, empowerment, and satisfaction among fintech employees. BMC Psychol. 13:3104. doi: 10.1186/s40359-025-03104-1, 40635061 PMC12243145

[ref44] Loghmani M. Webb T. Cuskelly G. Alavi S. H. (2023). How job crafting builds organizational agility in a government-dependent NSO: the mediating role of organizational climate. Manag. Sport Leis. 28, 522–537. doi: 10.1080/23750472.2021.1937286

[ref45] Ludviga I. Kalvina A. (2024). Organizational agility during crisis: do employees’ perceptions of public sector organizations’ strategic agility Foster employees’ work engagement and well-being? Empl. Responsib. Rights J. 36, 1–21. doi: 10.1007/s10672-023-09442-9, 40479364 PMC9988600

[ref46] Maran T. K. Liegl S. Davila A. Moder S. Kraus S. Mahto R. V. (2022). Who fits into the digital workplace? Mapping digital self-efficacy and agility onto psychological traits. Technol. Forecast. Soc. Change 175:121352. doi: 10.1016/j.techfore.2021.121352

[ref48] Muduli A. (2016). Exploring the facilitators and mediators of workforce agility: an empirical study. Manag. Res. Rev. 39, 1567–1586. doi: 10.1108/MRR-10-2015-0236

[ref49] Muduli A. (2017). Workforce agility: examining the role of organizational practices and psychological empowerment. Glob. Bus. Organ. Excel. 36, 46–56. doi: 10.1002/joe.21800

[ref9009] Muduli A. Choudhury A. (2024). Digital technology adoption, workforce agility and digital technology outcomes in the context of the banking industry of India. Journal of Science and Technology Policy Management. doi: 10.1108/JSTPM-01-2024-0018

[ref50] Muduli A. Choudhury A. (2025). Exploring the role of workforce agility on digital transformation: a systematic literature review. Benchmarking 32, 492–512. doi: 10.1108/BIJ-02-2023-0108

[ref51] Muduli A. Pandya G. (2018). Psychological empowerment and workforce agility. Psychol. Stud. 63, 276–285. doi: 10.1007/s12646-018-0456-8

[ref52] Musaigwa M. (2023). The role of leadership in managing change. Int. Rev. Manag. Mark. 13, 1–9. doi: 10.32479/irmm.13526

[ref53] Naim M. F. Sahai S. Elembilassery V. (2024). Does empowering leadership enhance employee agility? A serial mediation model. Evidence-Based HRM Global Forum Emp. Scholarship 12, 666–682. doi: 10.1108/EBHRM-08-2022-0197

[ref55] Neumann O. Kirklies P. C. Schott C. (2024). Adopting agile in government: a comparative case study. Public Manag. Rev. 26, 3692–3714. doi: 10.1080/14719037.2024.2354776

[ref54] Neumann O. Kirklies P.-C. Hadorn S. (2024). Does agile improve value creation in government? Inf. Polity 29, 235–252. doi: 10.3233/ip-230060

[ref56] Nguyen T. Le C. V. Nguyen M. Nguyen G. Lien T. T. H. Nguyen O. (2024). The organisational impact of agility: a systematic literature review. Manag. Rev. Q. 75:446. doi: 10.1007/s11301-024-00446-9

[ref57] Öztürk, O., Kocaman R. Kanbach D. K. (2024). How to design bibliometric research: an overview and a framework proposal. Rev. Manag. Sci., 18, 3333–3361. doi: 10.1007/s11846-024-00738-0

[ref58] Panda P. Singh P. (2025a). Organizational virtuousness fosters innovative performance and pro-social behavior: examining the role of. J. Organ. Chang. Manag. 38, 701–721. doi: 10.1108/JOCM-02-2024-0073

[ref59] Panda P. Singh P. (2025b). Perceived organizational virtuousness and employee’ s subjective well-being: examining the role of resilience, agility. Manag. Decis. doi: 10.1108/MD-01-2024-0076

[ref60] Panda P. Singh P. (2026). Resilient and agile employees’ pursuit of innovative performance and well-being: the role of job crafting. Global Knowledge Mem. Commun. 75, 300–315. doi: 10.1108/GKMC-11-2023-0450

[ref61] Passas I. (2024). Bibliometric analysis: the main steps. Encyclopedia 4, 1014–1025. doi: 10.3390/encyclopedia4020065

[ref62] Petermann M. K. H. Zacher H. (2020). Agility in the workplace: conceptual analysis, contributing factors, and practical examples. Ind. Organ. Psychol. 13, 599–609. doi: 10.1017/iop.2020.106

[ref63] Petermann M. K. H. Zacher H. (2021). Development of a behavioral taxonomy of agility in the workplace. Int. J. Manag. Proj. Bus. 14, 1383–1405. doi: 10.1108/IJMPB-02-2021-0051

[ref9010] Petermann M. K. H. Zacher H. (2022). Workforce agility: development and validation of a multidimensional measure. Front. Psychol. 13. doi: 10.3389/fpsyg.2022.841862 PMC8992541 35401298

[ref64] Pitafi A. H. (2025). Enterprise social media as enablers of employees’ agility: the impact of work stress and enterprise social media visibility. Inf. Technol. People 38, 1230–1253. doi: 10.1108/ITP-10-2022-0791

[ref65] Pitafi A. H. Albishri N. Chotia V. Islam N. Sahore N. (2025a). Transforming agility performance in enterprise information management: the impact of ESM interactivity and communication visibility. J. Enterp. Inf. Manag. 38:15. doi: 10.1108/JEIM-01-2025-0015

[ref71] Pitafi A. H. Ren M. (2021). Predicting the factors of employee agility using enterprise social media: moderating effects of enterprise social media-related strain. Internet Res. 31, 1963–1990. doi: 10.1108/INTR-11-2019-0469

[ref66] Pitafi A. H. Islam N. Basahel S. Attri R. Singhal A. B. (2025b). Enterprise social media and employee agility: the role of task context and personal motivation. IEEE Trans. Eng. Manag. 72, 3305–3317. doi: 10.1109/TEM.2025.3587594

[ref67] Pitafi A. H. Kanwal S. Pitafi A. (2019). Effect of enterprise social media and psychological safety on employee’s agility: mediating role of communication quality. Int. J. Agile Syst. Manag. 12, 1–26. doi: 10.1504/IJASM.2019.098708

[ref68] Pitafi A. H. Liu H. Cai Z. (2018). Investigating the relationship between workplace conflict and employee agility: the role of enterprise social media highlights. Telematics Inform. doi: 10.1016/j.tele.2018.08.001

[ref69] Pitafi A. H. Masood F. Pitafi S. (2025c). Unlocking potential: how enterprise social media features shape employee agility performance. Inf. Technol. People. doi: 10.1108/ITP-01-2024-0064

[ref70] Pitafi A. H. Rasheed M. I. Kanwal S. Ren M. (2020). Employee agility and enterprise social media: the role of IT proficiency and work expertise. Technol. Soc. 63:101333. doi: 10.1016/j.techsoc.2020.101333

[ref72] Pitafi A. H. Yaqub M. Z. Gupta P. Alzeiby E. A. Fiano F. (2025d). Investigating the impact of work stress and knowledge transfer on employee agility performance: work-related ESM usage as a moderator. J. Knowl. Manag. 29, 1561–1593. doi: 10.1108/JKM-05-2024-0520

[ref3001] Prieto L. Talukder M. F. (2023). Resilient Agility: A Necessary Condition for Employee and Organizational Sustainability. Sustainability (Switzerland), 15. doi: 10.3390/su15021552

[ref73] Rasheed M. I. Pitafi A. H. (2025). Task structure and knowledge transfer: leveraging employee agility performance in an ESM environment. Behav. Inf. Technol. 44, 1925–1941. doi: 10.1080/0144929X.2024.2383260

[ref74] Rasheed M. I. Pitafi A. H. Mishra S. Chotia V. (2023). When and how ESM affects creativity: the role of communication visibility and employee agility in a cross-cultural setting. Technol. Forecast. Soc. Change 194:122717. doi: 10.1016/j.techfore.2023.122717

[ref75] Rastogi S. Pandita D. (2025). Driving entrepreneurial success: navigating AI-driven transformation through workforce agility and sustainability. J. Innov. Entrepren. 14:554. doi: 10.1186/s13731-025-00554-0

[ref76] Rauf A. Jin Z. Roshi E. E. Naseer S. Khalid S. Parveen S. . (2023). COVID-19 outbreak: impact on global economy. Front. Public Health 5, 1–13. doi: 10.3389/fpubh.2022.1009393 PMC9923118 36793360

[ref77] Raut P. K. Das J. R. Gochhayat J. Das K. P. (2022). Influence of workforce agility on crisis management: role of job characteristics and higher administrative support in public administration. Mater. Today Proc. 61, 647–652. doi: 10.1016/j.matpr.2021.08.121

[ref9012] Reyes L.-E. Blanco M.-R. Pinillos M.-J. (2024). Past, present, and future of learning agility: a bibliometric and content analysis. Hum. Resour. Dev. Rev. 23, 462–497. doi: 10.1177/15344843241258522

[ref9013] Salmen K. Festing M. (2022). Paving the way for progress in employee agility research: a systematic literature review and framework. Int. J. Hum. Resour. Manag. 33, 4386–4439. doi: 10.1080/09585192.2021.1943491

[ref78] Sameer S. K. (2024). The interplay of digitalization, organizational support, workforce agility and task performance in a blended working environment: evidence from Indian public sector organizations. Asian Bus. Manag. 23, 1–21. doi: 10.1057/s41291-022-00205-2, 40479235 PMC9574795

[ref79] Selznick P. (1948). Foundations of the theory of organization. Am. Sociol. Rev. 13:25. doi: 10.2307/2086752

[ref80] Sharma K. Nigam N. Jha J. K. Xu X. (2024). Role of readiness to change in the relationship between workforce agility and digital transformation: a two-timeframe study. J. Glob. Inf. Manag. 32:241. doi: 10.4018/JGIM.345241

[ref9014] Sherehiy B. Karwowski W. (2014). The relationship between work organization and workforce agility in small manufacturing enterprises. Int. J. Ind. Ergon. 44, 466–473. doi: 10.1016/j.ergon.2014.01.002

[ref81] Sony M. Mekoth N. (2012). A typology for frontline employee adaptability to gain insights in service customisation: a viewpoint Michael Sony * Nandakumar Mekoth. Int. J. Serv. Oper. Manag. 12, 490–508.

[ref82] Sony M. Mekoth N. (2014). The dimensions of frontline employee adaptability in power sector a grounded theory approach. Int. J. Energy Sect. Manag. 8, 240–258. doi: 10.1108/IJESM-03-2013-0008

[ref83] Sony M. Mekoth N. (2015). Fleadapt scale: a new tool to measure frontline employee adaptability in power sector. Int. J. Energy Sect. Manag. 9, 496–522. doi: 10.1108/ijesm-05-2014-0005

[ref84] Sony M. Mekoth N. (2016a). Journal of retailing and consumer services the relationship between emotional intelligence, frontline employee adaptability, job satisfaction and job performance. J. Retail. Consum. Serv. 30, 20–32. doi: 10.1016/j.jretconser.2015.12.003

[ref85] Sony M. Mekoth N. (2017a). The mediation role of frontline employee adaptability between service orientation and job outcomes: evidence from Indian power sector. Int. J. Bus. Excel. 11, 357–380. doi: 10.1504/IJBEX.2017.081931

[ref86] Sony M. Mekoth N. (2017b). Workplace spirituality, frontline employee adaptability and job outcomes: an empirical study. Int. J. Process Manag. Benchmarking 7, 437–465. doi: 10.1504/IJPMB.2017.086925

[ref87] Sony M. Mekoth N. (2019). Broadening the lean six sigma concept through employee adaptability: a literature review. Int. J. Prod. Q. 28:3. doi: 10.1504/IJPQM.2019.103522

[ref88] Sony M. Mekoth N. (2022b). Employee adaptability skills for industry 4.0 success: a road map. Production & Manufacturing Research 10, 24–41. doi: 10.1080/21693277.2022.2035281

[ref89] Srigouri V. V. Muduli A. (2024a). Performance coaching and training transfer in micro, small and medium enterprises of India: examining the mediating role of employee agility. J. Manage. Dev. 43, 556–570. doi: 10.1108/JMD-12-2023-0360

[ref90] Srigouri V. V. Muduli A. (2024b). Training transfer in MSMEs of India: examining the role of performance coaching, HRD climate and employee agility. Ind. Commer. Training 56, 419–433. doi: 10.1108/ICT-05-2024-0043

[ref91] Srivastava S. Gupta P. (2022). Workplace spirituality as panacea for waning well-being during the pandemic crisis: a SDT perspective. J. Hosp. Tour. Manag. 50, 375–388. doi: 10.1016/j.jhtm.2021.11.014

[ref92] Storme M. Suleyman O. Gotlib M. Lubart T. (2020). Who is agile? An investigation of the psychological antecedents of workforce agility. Glob. Bus. Organ. Excel. 39, 28–38. doi: 10.1002/joe.22055

[ref93] Troise C. Corvello V. Ghobadian A. Regan N. O. (2022). How can SMEs successfully navigate VUCA environment: the role of agility in the digital transformation era. Technol. Forecast. Soc. Change 174:121227. doi: 10.1016/j.techfore.2021.121227

[ref94] Vapiwala F. Rastogi S. Pandita D. (2025). Is workforce agility the new agenda? Perspectives on the role of constructive task conflicts. Employee Responsib. Rights J. doi: 10.1007/s10672-025-09528-6

[ref95] Varshney D. Varshney N. K. (2024). Does empowering leadership behavior affect employee performance? The mediating role of workforce agility. Int. J. Prod. Perform. Manag. 74, 1425–1451. doi: 10.1108/IJPPM-11-2023-0618

[ref9016] Walter A. T. (2021). Organizational agility: ill-defined and somewhat confusing? A systematic literature review and conceptualization. In Management review quarterly (Springer International Publishing. Vol. 71. doi: 10.1007/s11301-020-00186-6

[ref96] Wang H.-J. Demerouti E. Le Blanc P. (2017). Transformational leadership, adaptability, and job crafting: the moderating role of organizational identification. J. Vocat. Behav. 100, 185–195. doi: 10.1016/j.jvb.2017.03.009

[ref97] Weber E. Büttgen M. Bartsch S. (2022). How to take employees on the digital transformation journey: an experimental study on complementary leadership behaviors in managing organizational change. J. Bus. Res. 143, 225–238. doi: 10.1016/j.jbusres.2022.01.036

[ref98] Wei C. Pitafi A. H. Kanwal S. Ali A. Ren M. (2020). Improving employee agility using enterprise social media and digital fluency: moderated mediation model. IEEE Access 8, 68799–68810. doi: 10.1109/ACCESS.2020.2983480

[ref99] Yesudhas D. Srivastava A. Gromiha M. M. (2021). COVID-19 outbreak: history, mechanism, transmission, structural studies and therapeutics. Infection 49, 199–213. doi: 10.1007/s15010-020-01516-2, 32886331 PMC7472674

[ref100] Zhang J. Li H. Zhao H. (2025). The impact of digital transformation on organizational resilience: the role of innovation capability and agile response. Systems 13, 1–26. doi: 10.3390/systems13020075

[ref101] Zhang L. Xu Y. Chen C. Zhao R. (2022). Predicting the factors of employee agility using enterprise social media: the moderating role of innovation culture. Front. Psychol. 13, 1–11. doi: 10.3389/fpsyg.2022.911427, 35814160 PMC9263975

[ref103] Zhang S. Suntrayuth S. (2024). The synergy of ambidextrous leadership, agility, and entrepreneurial orientation to achieve sustainable AI product innovation. Sustainability 16:248. doi: 10.3390/su16104248

[ref104] Zhu Y. Quansah P. E. Obeng A. F. Minyu G. (2023). High-performance work systems and safety performance in the mining sector: exploring the mediating influence of workforce agility and moderating effect of safety locus of control. Curr. Psychol. 42, 25100–25126. doi: 10.1007/s12144-022-03606-w

